# New distributional records of fireflies (Coleoptera, Lampyridae, Luciolinae) from two Eastern States of India with notes on their biology and an updated Indian checklist

**DOI:** 10.3897/BDJ.11.e98948

**Published:** 2023-03-24

**Authors:** Srinjana Ghosh, Subhankar Kumar Sarkar, Susanta Chakraborty

**Affiliations:** 1 Department of Zoology, Bethune College, Kolkata, India Department of Zoology, Bethune College Kolkata India; 2 Entomology Laboratory, Department of Zoology, University of Kalyani, Kalyani, India Entomology Laboratory, Department of Zoology, University of Kalyani Kalyani India; 3 Department of Zoology, Vidyasagar University, Midnapore, India Department of Zoology, Vidyasagar University Midnapore India

**Keywords:** Asia, distribution, flashing firefly, Oriental, taxonomy

## Abstract

**Background:**

The subfamily Luciolinae Lacordaire, 1857, a group of flashing fireflies, is composed of approximately 400 described species in the world. Though the taxonomy of this group has been fairly well established in Southeast (SE) Asia and the Australopacific Region, there is scope of gathering further information regarding taxonomic knowledge on this group from India. Until now, 32 Luciolinae species from nine genera have been reported from India, which is only about 8% (32 out of 400) of the world Luciolinae fauna. With this in mind, several faunistic surveys were conducted in Odisha and West Bengal States of India to explore the firefly fauna of the two States.

**New information:**

The faunistic surveys conducted in the Odisha and West Bengal States of India have revealed six new distributional records of Luciolinae firefly species belonging to the genera *Abscondita* Ballantyne, Lambkin & Fu 2013, *Asymmetricata* Ballantyne 2009, *Pteroptyx* Olivier 1902 and *Sclerotia* Ballantyne 2016 in the two States, earlier which were recorded from other States of India. Of the already recorded species, *Abs.perplexa* is recorded for the first time from both the States, whereas *Abs.chinensis*, *Asy.ovalis* and *Scl.aquatilis* present new records for Odisha State and *Pt.malaccae* and *Scl.substriata* for West Bengal State. The newly-recorded taxa are presented with their examined materials, diagnoses and brief biological notes. Some more distinguishing characters were added for the male genitalia of *Abs.perplexa* and *Asy.ovalis*. Further, a checklist of Luciolinae firefly species already recorded from India is also presented.

## Introduction

The family Lampyridae Rafinesque, 1815 is composed of approximately 2400 species distributed over 144 genera and 12 subfamilies (McDermott 1966, Branham 2010, Martin et al. 2017, Martin et al. 2019, Ferreira et al. 2019, Ferreira et al. 2020, Bocakova et al. 2022). Of these, the subfamily Luciolinae Lacordaire, 1857 alone consists of more than 400 described species distributed over 32 genera (Ballantyne et al. 2015, Ballantyne et al. 2016, Ballantyne et al. 2019). The Southeast (SE) Asia and the Australopacific Region possess a remarkable asset of Lucioline species (~ 303 species) (Ballantyne et al. 2019). Countries in Southeast Asia have a high firefly biodiversity (Ballantyne et al. 2019, Jusoh et al. 2021).

India, though it is recognised as one of the mega-diverse countries of the world, its firefly fauna is very much understudied and has not received much attention for the last 100 years or so. Until now, only 32 Luciolinae species belonging to genera *Abscondita* Ballantyne Lambkin & Fu, 2013 (three species), *Asymmetricata* Ballantyne, 2009 (three species), *Curtos* Motschulsky, 1854 (two species), *Inflata* Boontop, 2015 (one species), *Luciola* Laporte, 1833 (15 species), *Pteroptyx* Olivier, 1902 (one species), *Pygoluciola* Wittmer, 1939 (four species), *Pyrophanes* Olivier, 1883 (one species) and *Sclerotia* Ballantyne, 2016 (two species) have been reported from India (Gorham 1895, Gorham 1903, McDermott 1966, Ballantyne et al. 2013, Ballantyne et al. 2016, Ballantyne et al. 2019, Ghosh et al. 2021). Of these, *Abs.chinensis*, *Abs.terminalis*, *Asy.ovalis*, *L.gigas*, *L.pallidipes*, *Py.nitescens*, *Py.vitalisi*, and *Scl.aquatilis* have been reported from the State of West Bengal, while only *Abs.terminalis* has been reported from the State of Odisha until now (Gorham 1880, McDermott 1966, Ballantyne et al. 2019, Ghosh et al. 2021).

It is with this background that several faunistic surveys for Luciolinae fireflies have been conducted in two eastern States of India (Odisha and West Bengal) during February 2017 to March 2020. The survey results also confirmed the further occurrence of the above-mentioned species in India and in these two States. Additionally, *Abs.perplexa* is recorded for the first time from both States, whereas *Abs.chinensis*, *Asy.ovalis* and *Scl.aquatilis* present new records for Odisha State and *Pt.malaccae* and *Scl.substriata* for West Bengal State. Until now, *Abs.perplexa* had records from Assam and Madhya Pradesh States, *Abs.chinensis* from Assam, Bihar, Maharashtra and West Bengal States, *Asy.ovalis* from Goa and West Bengal States, *Pt.malaccae* from Tamilnadu State, *Scl.aquatilis* from Maharashtra and West Bengal States and *Scl.substriata* from Assam and Maharashtra States (Gorham 1880, Ballantyne et al. 2013, Ballantyne et al. 2016, Ballantyne et al. 2019).

Moreover, an Indian checklist (Table 1) of Luciolinae fireflies is also compiled mostly on the basis of Ballantyne et al. (2019) and all other available literature including the publications (Das and Dev Roy 1989, Sengupta and Mukhopadhyay 1990, Kacker 1993, Agarwal and Ghose 1995a, Agarwal and Ghose 1995b, Agarwal and Ghose 1995c, Majumder and Dey 2005, Mitra 2005, Roy and Nandi 2012) of the Zoological Survey of India (ZSI) and the records (Kapur 1955) of the Indian Museum.

## Materials and methods

### Study sites

**Figure 1.**
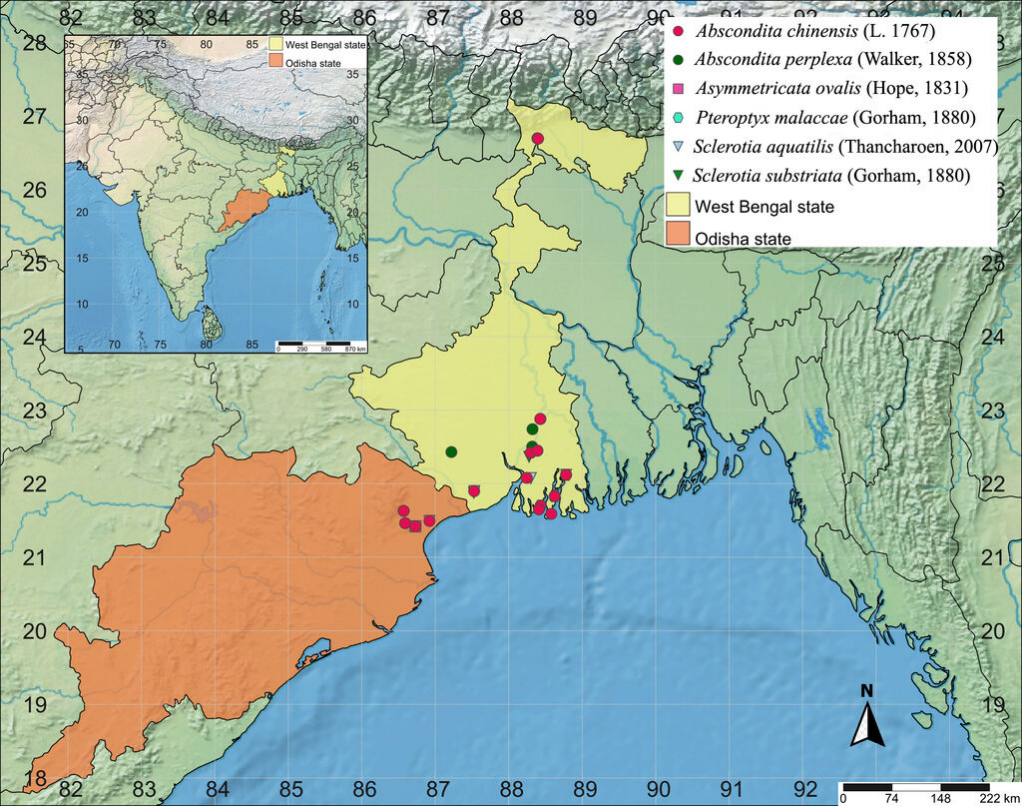
Map of the study area indicating collection points of newly-recorded species (India in inset highlighting the two eastern States).

The surveys were carried out in selected study sites of the two eastern States of India (Fig. 1), representing contrasting characteristics with respect to habitat, ecological attributes and anthropogenic interaction types and levels. The study sites are:

**Tropical moist deciduous forest of Kuldiha Wildlife Sanctuary and its associated areas.** Located in the Balasore District of Odisha State, the site is spread over an area of around 272.75 km^2^ and exhibits a typical mixture of semi-evergreen riparian forests, peninsular (coastal) Sal Forest (*Shorearobusta*) and tropical moist deciduous forest.

**Coastal zone of Chandipore.** Situated along the Bay of Bengal in the northern part of the east coast of India in the Balasore District of Odisha State, the site represents typical marshy tidal flat with sandy alluvial soil type.

**Mixed dry deciduous vegetation patch of Keonjhar.** The site exhibits tropical mixed dry deciduous vegetation with underneath granite rocks and its metamorphs consisting of significant mineral assets like iron ore, manganese and chromites.

**Himalayan foothills of Darjeeling.** The site has a characterised substratum with sandstone and conglomerate formations, horizontal layers of unconsolidated sand, silt, pebbles and gravel, covered with tropical deciduous vegetation prominently represented by Sal trees.

**Southern Gangetic plains.** The site has deltaic alluvial soil characterised by evergreen vegetation and prevalent humid climate.

**Mangrove forests of Sundarban Biosphere Reserve.** The site represents various islands having typical mangrove patches and areas with reclaimed land forms.

**Gurguripal Eco Forest.** The site is a forest-based rural area with highly eroded red lateritic soil with tropical evergreen and mixed deciduous Sal Forest.

**Coastal alluvial plain of Egra.** Situated in the lower part of the coastal tract in West Bengal, the area is covered by alluvial plains with scarcely distributed halophytic vegetation.

### Specimen collection and identification

The specimens were collected through random and selective (display plant based) net sweeping and hand-picking techniques, guided mainly by their luminescent display. Specimens after collection were preserved in 70% ethanol in glass vials. Species identification was done following the keys of Ballantyne and Lambkin (2009), Ballantyne and Lambkin (2013), Ballantyne et al. (2016), Ballantyne et al. (2019), Jusoh et al. (2018) and Jusoh et al. (2021). Photographs were captured by wi-fi digital microscope (Tobo) and CMOS camera (MAGCAM DC5) attached to a stereozoom trinocular microscope (OLYMPUS SZX7). The abbreviations and terminologies used in this work are based on Ballantyne and Lambkin (2013), Ballantyne et al. (2013), Ballantyne et al. (2016) and Ballantyne et al. (2019) and are as follows: ASD – distance between antennal sockets; ASW – antennal socket greatest diameter; BP – Basal piece of aedeagus; GHW – greatest head width (across eyes, measured parallel to ASD); L – Length; LL – Lateral lobes of aedeagus; LO – Light organs; ML – Median lobe of aedeagus; MN – Mesonotum; MPP – Median posterior projection of ventrite 7, male only; MN – Mesonotum; MS – Mesoscutellum; PLP – Posterolateral projection of Ventrite 7, male only; PN – Pronotum; T7, T8 etc. – Visible abdominal tergites; V3,V4,….V7 etc. – Abdominal ventrites, referred to by actual, not visible numbers; W – Width; a, b – aedeagal dimensions (a = distance from dorsal base of lateral lobes to apex of median lobe, b = distance from dorsal base of lateral lobes to apex of lateral lobes, expressed as b/a).

### Material deposition

The specimens are deposited in the entomological collections of the Ecology Laboratory of the Department of Zoology, Vidyasagar University (VUEC), Midnapore (West), West Bengal, India.

## Taxon treatments

### Abscondita chinensis

(L., 1767)

BEB57D66-3523-5752-9C9E-163FC92CF50A

#### Materials

- **Type status:** Other material. **Occurrence:** recordedBy: Srinjana Ghosh; sex: 2 males, 4 females; occurrenceID: AB0E20C1-3457-5C1B-9559-3082E7AEDB29; **Taxon:** scientificName: *Absconditachinensis* (L., 1767); family: Lampyridae; **Location:** country: India; countryCode: IND; stateProvince: Odisha; locality: Kuldiha Wildlife Sanctuary; verbatimCoordinates: 21°25'12''N, 86°43'48''E; **Event:** samplingProtocol: Sweep net; eventDate: 24-April-17; **Record Level:** collectionCode: VUEC-0002, VUEC-0003, VUEC-0004, VUEC-0005, VUEC-0006, VUEC-0007; basisOfRecord: Preserved specimen
- **Type status:** Other material. **Occurrence:** recordedBy: Srinjana Ghosh; sex: 1 male, 6 females; occurrenceID: AA35C61D-0225-51DD-AA9E-5EE08EEA9701; **Taxon:** scientificName: *Absconditachinensis* (L., 1767); family: Lampyridae; **Location:** country: India; countryCode: IND; stateProvince: Odisha; locality: Area near Rissia Dam adjacent to Kuldiha Wildlife Sanctuary; verbatimCoordinates: 21°27'46''N, 86°35'30''E; **Event:** samplingProtocol: Hand picking; eventDate: 27-April-17; **Record Level:** collectionCode: VUEC-0008, VUEC-0009, VUEC-0010, VUEC-0011, VUEC-0012, VUEC-0013, VUEC-0014; basisOfRecord: Preserved specimen
- **Type status:** Other material. **Occurrence:** recordedBy: Srinjana Ghosh; sex: 3 males; occurrenceID: 903426A8-D149-55CF-820E-2AC20FB6DE6F; **Taxon:** scientificName: *Absconditachinensis* (L., 1767); family: Lampyridae; **Location:** country: India; countryCode: IND; stateProvince: West Bengal; locality: area near Sajnekhali Bird Sanctuary, South 24 Pargana; verbatimCoordinates: 22°7'12''N, 88°46'49''E; **Event:** samplingProtocol: Hand picking; eventDate: 7-July-17; **Record Level:** collectionCode: VUEC-0022, VUEC-0023, VUEC-0024; basisOfRecord: Preserved specimen
- **Type status:** Other material. **Occurrence:** recordedBy: Srinjana Ghosh; sex: 2 maless; occurrenceID: E6A23DBE-2E55-5D13-9339-BAC0EDA1C5B2; **Taxon:** scientificName: *Absconditachinensis* (L., 1767); family: Lampyridae; **Location:** country: India; countryCode: IND; stateProvince: West Bengal; locality: Egra, East Midnapor; verbatimCoordinates: 21°53'56''N, 87°31'48''E; **Event:** samplingProtocol: Hand picking; eventDate: 11-February-18; **Record Level:** collectionCode: VUEC-0028, VUEC-0029; basisOfRecord: Preserved specimen
- **Type status:** Other material. **Occurrence:** recordedBy: Srinjana Ghosh; sex: 2 males; occurrenceID: 57CA9944-BFE0-5BB7-AC86-AB4D2F5B0292; **Taxon:** scientificName: *Absconditachinensis* (L., 1767); family: Lampyridae; **Location:** country: India; countryCode: IND; stateProvince: West Bengal; locality: Sonarpur, South 24 Pargana; verbatimCoordinates: 22°26'57''N, 88°23'25''E; **Event:** samplingProtocol: Hand picking, Net sweeping; eventDate: 11-March-18; **Record Level:** collectionCode: VUEC-0030, VUEC-0031; basisOfRecord: Preserved specimen
- **Type status:** Other material. **Occurrence:** recordedBy: Srinjana Ghosh; sex: 1 male; occurrenceID: A2FD83B4-8675-54D4-8ECD-9F1D16C228A9; **Taxon:** scientificName: *Absconditachinensis* (L., 1767); family: Lampyridae; **Location:** country: India; countryCode: IND; stateProvince: West Bengal; locality: Indrapore village, South 24 Pargana; verbatimCoordinates: 21°39'00''N, 88°24'36''E; **Event:** samplingProtocol: Hand picking,; eventDate: 15-March-18; **Record Level:** collectionCode: VUEC-0033; basisOfRecord: Preserved specimen
- **Type status:** Other material. **Occurrence:** recordedBy: Srinjana Ghosh; sex: 4 males; occurrenceID: 49E7C22A-2DBF-5D84-97F1-B80C2453E2F8; **Taxon:** scientificName: *Absconditachinensis* (L., 1767); family: Lampyridae; **Location:** country: India; countryCode: IND; stateProvince: West Bengal; locality: Enayetpur, Kulpi, South 24 Parganas; verbatimCoordinates: 22°04'44''N, 88°14'42''E; **Event:** samplingProtocol: Hand picking; eventDate: 3-May-18; **Record Level:** collectionCode: VUEC-0036, VUEC-0037, VUEC-0038, VUEC-0039; basisOfRecord: Preserved specimen
- **Type status:** Other material. **Occurrence:** recordedBy: Srinjana Ghosh; sex: 2 males;; occurrenceID: D420B15D-41BA-5D37-8904-061AF251FA89; **Taxon:** scientificName: *Absconditachinensis* (L., 1767); family: Lampyridae; **Location:** country: India; countryCode: IND; stateProvince: Odisha; locality: near Chandipore coast, Balasore; verbatimCoordinates: 21°29'36'' N, 86°55'19''E; **Event:** samplingProtocol: Hand picking; eventDate: 8-May-18; **Record Level:** collectionCode: VUEC-0040, VUEC-0041; basisOfRecord: Preserved specimen
- **Type status:** Other material. **Occurrence:** recordedBy: Srinjana Ghosh; sex: 2 males; occurrenceID: CAE05A54-5AEC-5A0E-9BC0-C9F33802AE87; **Taxon:** scientificName: *Absconditachinensis* (L., 1767); family: Lampyridae; **Location:** country: India; countryCode: IND; stateProvince: West Bengal; locality: Kawakhali village, Darjeeling; verbatimCoordinates: 26°41'41''N, 88°23'28''E; **Event:** samplingProtocol: Net sweeping; eventDate: 15-July-18; **Record Level:** collectionCode: VUEC-0057, VUEC-0058; basisOfRecord: Preserved specimen
- **Type status:** Other material. **Occurrence:** recordedBy: Srinjana Ghosh; sex: 2 males; occurrenceID: AA254A00-E2F3-5443-80A6-DCE0566D1028; **Taxon:** scientificName: *Absconditachinensis* (L., 1767); family: Lampyridae; **Location:** country: India; countryCode: IND; stateProvince: West Bengal; locality: North Bengal University Campus, Darjeeling; verbatimCoordinates: 26°41'24''N, 88°23'59''E; **Event:** samplingProtocol: Net sweeping; eventDate: 16-July-18; **Record Level:** collectionCode: VUEC-0061, VUEC-0062; basisOfRecord: Preserved specimen
- **Type status:** Other material. **Occurrence:** recordedBy: Srinjana Ghosh; sex: 4 males; occurrenceID: E26D41E7-BCEC-56A4-9574-42284D9FB059; **Taxon:** scientificName: *Absconditachinensis* (L., 1767); family: Lampyridae; **Location:** country: India; countryCode: IND; stateProvince: West Bengal; locality: Naihati, North 24 Parganas; verbatimCoordinates: 22°52'48''N, 88°25'48''E; **Event:** samplingProtocol: Net sweeping; eventDate: 25-August-18; **Record Level:** collectionCode: VUEC-0066, VUEC-0067, VUEC-0068, VUEC-0069; basisOfRecord: Preserved specimen
- **Type status:** Other material. **Occurrence:** recordedBy: Srinjana Ghosh; sex: 5 males; occurrenceID: 13F07B37-3990-5D5D-B509-45517EEB941A; **Taxon:** scientificName: *Absconditachinensis* (L., 1767); family: Lampyridae; **Location:** country: India; countryCode: IND; stateProvince: West Bengal; locality: Pailan, South 24 Parganas; verbatimCoordinates: 22°25'12''N, 88°17'59''E; **Event:** samplingProtocol: Hand picking; eventDate: 22-February-19; **Record Level:** collectionCode: VUEC-0071, VUEC-0072, VUEC-0073, VUEC-0074, VUEC-0075; basisOfRecord: Preserved specimen
- **Type status:** Other material. **Occurrence:** recordedBy: Srinjana Ghosh; sex: 2 males; occurrenceID: 7299EACE-E4AA-57B9-97D5-0D70D6FF5A02; **Taxon:** scientificName: *Absconditachinensis* (L., 1767); family: Lampyridae; **Location:** country: India; countryCode: IND; stateProvince: West Bengal; locality: Kalash Island, South 24 Parganas; verbatimCoordinates: 21°35'24''N, 88°34'50''E; **Event:** samplingProtocol: Hand picking; eventDate: 15-March-19; **Record Level:** collectionCode: VUEC-0082, VUEC-0083; basisOfRecord: Preserved specimen
- **Type status:** Other material. **Occurrence:** recordedBy: Srinjana Ghosh; sex: 2 males; occurrenceID: 61900470-36F7-5008-A6F8-8B0B393CC555; **Taxon:** scientificName: *Absconditachinensis* (L., 1767); family: Lampyridae; **Location:** country: India; countryCode: IND; stateProvince: West Bengal; locality: Bonnie Island, South 24 Parganas; verbatimCoordinates: 21°49'50''N, 88°37'26''E; **Event:** samplingProtocol: Hand picking; eventDate: 17-March-19; **Record Level:** collectionCode: VUEC-0084, VUEC-0085; basisOfRecord: Preserved specimen
- **Type status:** Other material. **Occurrence:** recordedBy: Srinjana Ghosh; sex: 2 males; occurrenceID: E3B0AE9D-3CF3-5953-8FFB-A9CF0A994424; **Taxon:** scientificName: *Absconditachinensis* (L., 1767); family: Lampyridae; **Location:** country: India; countryCode: IND; stateProvince: West Bengal; locality: Dhainchi Island, South 24 Parganas; verbatimCoordinates: 21°42' 05''N, 88°26'00''E; **Event:** samplingProtocol: Hand picking; eventDate: 18-March-19; **Record Level:** collectionCode: VUEC-0086, VUEC-0087; basisOfRecord: Preserved specimen
- **Type status:** Other material. **Occurrence:** recordedBy: Srinjana Ghosh; sex: 2 males, 2 females; occurrenceID: BE06C6E0-3C5B-5DE0-A584-92ACD3CE7290; **Taxon:** scientificName: *Absconditachinensis* (L., 1767); family: Lampyridae; **Location:** country: India; countryCode: IND; stateProvince: Odisha; locality: Judia, Keonjhar; verbatimCoordinates: 21°37'48''N, 86°34'14''E; **Event:** samplingProtocol: Hand picking; eventDate: 24- March-19; **Record Level:** collectionCode: VUEC-0092, VUEC-0093, VUEC-0094, VUEC-0095; basisOfRecord: Preserved specimen

#### Diagnosis

Length 8–10 mm; Width 2.8–3.2 mm; *Abs.chinensis* is one of the three species of *Abscondita* (*Abs.chinensis*, *Abs.perplexa* and *Abs.terminalis*) present in India, which is characterised by the pale dorsum with dark brown to black elytral apices (Fig. 2). PN, MS, ventral thorax and elytra pale brown with the elytral apices black and occupying approximately 1/6 of its total length, MN pale yellow, antennae and palpi dark brown, terminal abdominal tergites dark brown, ventral abdomen brownish-yellow, white LO on V6 and V7, legs brownish-yellow with the tips of tibae and tarsi dark brown. The species can be distinguished from other *Abscondita* species known from India by the following set of male characters: completely dark brown V5 (in *Abs.perplexa* and *Abs.terminalis*, the V5 is partly dark along the posterior margin only). Male genitalia (Fig. 3): Aedeagal sheath sternite terminated into two apically acute divergent lobes (in *Abs.perplexa* and *Abs.terminalis*, aedeagal sheath sternite is terminated into two apically rounded lobes); ML shorter than LL, gradually narrowed towards the apex, with the tips of LL blunt and extended beyond the tip of ML (in *Abs.perplexa*, both LL are longer than ML and the tip of ML is projecting beyond the tips of LL).

#### Biology

Adults of *Abs.chinensis* generally prefer open forested and grassland areas. These fireflies emit a luminescent flashing pattern, particularly during their mating season in early monsoon. During flight, males generally achieve an average height of 2 to 3 metres in forest and grassland habitat. Females answer to male courtship flashes from the substratum level or from the perching sites on ground vegetation. Bioluminescent larvae, which are terrestrial and inhabit places near aquatic bodies, show a carnivorous diet pattern and sometimes also cannibalism particularly when there is a scarcity of food (Ballantyne et al. 2013).

### Abscondita perplexa

(Walker, 1858)

E1AB208C-5371-5379-BE6E-B86FAFAEA309

#### Materials

- **Type status:** Other material. **Occurrence:** recordedBy: Srinjana Ghosh; sex: 3 males; occurrenceID: 2724AA64-FC90-5119-9559-B486898C7827; **Taxon:** scientificName: Absconditaperplexa (Walker, 1858); family: Lampyridae; **Location:** country: India; countryCode: Ind; stateProvince: Odisha; locality: area near Rissia Dam adjacent to Kuldiha Wildlife Sanctuary, Balasore; verbatimCoordinates: 21°27'46'' N, 86°35'30'' E; **Event:** samplingProtocol: Hand picking; eventDate: 26-April-17; **Record Level:** collectionCode: VUEC-0057, VUEC-0058, VUEC-0059; basisOfRecord: Preserved Specimen
- **Type status:** Other material. **Occurrence:** recordedBy: Srinjana Ghosh; sex: 1 male; occurrenceID: B5F02DD6-DB27-5834-8012-FA3DC9337607; **Taxon:** scientificName: Absconditaperplexa (Walker, 1858); family: Lampyridae; **Location:** country: India; countryCode: Ind; stateProvince: West Bengal; locality: Behala, South 24 Parganas; verbatimCoordinates: 22°30'23" N, 88°19'18" E; **Event:** samplingProtocol: Hand picking; eventDate: 22-June-17; **Record Level:** collectionCode: VUEC-0020; basisOfRecord: Preserved Specimen
- **Type status:** Other material. **Occurrence:** recordedBy: Srinjana Ghosh; sex: 1 male; occurrenceID: 56461559-3A80-5A56-B8E6-EC0E661F20C5; **Taxon:** scientificName: Absconditaperplexa (Walker, 1858); family: Lampyridae; **Location:** country: India; countryCode: Ind; stateProvince: West Bengal; locality: Sonarpur, South 24 Parganas; verbatimCoordinates: 22°26'57'' N, 88°23'25'' E; **Event:** samplingProtocol: Hand picking; eventDate: 11-March-18; **Record Level:** collectionCode: VUEC-0032; basisOfRecord: Preserved Specimen
- **Type status:** Other material. **Occurrence:** recordedBy: Srinjana Ghosh; sex: 1 male; occurrenceID: 4AD6E883-E83E-5B25-8341-248B7E4E715D; **Taxon:** scientificName: Absconditaperplexa (Walker, 1858); family: Lampyridae; **Location:** country: India; countryCode: Ind; stateProvince: West Bengal; locality: near Sajnekhali Bird Sanctuary, Sundarban Biosphere Reserve, South 24 Parganas; verbatimCoordinates: 22°7'12'' N, 88°46'49'' E; **Event:** samplingProtocol: Hand picking; eventDate: 16-March-18; **Record Level:** collectionCode: VUEC-0034; basisOfRecord: Preserved Specimen
- **Type status:** Other material. **Occurrence:** recordedBy: Santu Paria; sex: 1 male; occurrenceID: A165769B-07C9-56C6-B114-DB8E3EDBD4DE; **Taxon:** scientificName: Absconditaperplexa (Walker, 1858); family: Lampyridae; **Location:** country: India; countryCode: Ind; stateProvince: West Bengal; locality: Gurguripal Eco Forest, West Midnapore; verbatimCoordinates: 22°25'48'' N, 87°13'12'' E; **Event:** samplingProtocol: Hand picking; eventDate: 11-May-18; **Record Level:** collectionCode: VUEC-0043; basisOfRecord: Preserved Specimen
- **Type status:** Other material. **Occurrence:** recordedBy: Srinjana Ghosh; sex: 1 male; occurrenceID: 8D836B9B-7F0F-5BDF-A91E-939A67A1E359; **Taxon:** scientificName: Absconditaperplexa (Walker, 1858); family: Lampyridae; **Location:** country: India; countryCode: Ind; stateProvince: Odisha; locality: Kuldiha Wildlife Sanctuary, Balasore; verbatimCoordinates: 21°25'12''N, 86°43'48'' E; **Event:** samplingProtocol: Hand picking; eventDate: 27- May-18; **Record Level:** collectionCode: VUEC-0056; basisOfRecord: Preserved Specimen
- **Type status:** Other material. **Occurrence:** recordedBy: Srinjana Ghosh; sex: 1 male; occurrenceID: 78755CF3-8382-525A-A403-EA58B964C3C1; **Taxon:** scientificName: Absconditaperplexa (Walker, 1858); family: Lampyridae; **Location:** country: India; countryCode: Ind; stateProvince: West Bengal; locality: Shimla,Serampore, Hooghly; verbatimCoordinates: 22°44'27'' N, 88°19'24'' E; **Event:** samplingProtocol: Hand picking; eventDate: 1-July-18; **Record Level:** collectionCode: VUEC-0064; basisOfRecord: Preserved Specimen
- **Type status:** Other material. **Occurrence:** recordedBy: Srinjana Ghosh; sex: 1 male; occurrenceID: 7C5269D5-3EBA-529F-9839-2466E2AE452E; **Taxon:** scientificName: Absconditaperplexa (Walker, 1858); family: Lampyridae; **Location:** country: India; countryCode: Ind; stateProvince: West Bengal; locality: Kawakhali village, Darjeeling; verbatimCoordinates: 26°41'41'' N, 88°23'28'' E; **Event:** samplingProtocol: Hand picking; eventDate: 15-July-18; **Record Level:** collectionCode: VUEC-0065; basisOfRecord: Preserved Specimen
- **Type status:** Other material. **Occurrence:** recordedBy: Srinjana Ghosh; sex: 1 male; occurrenceID: E42CE528-C215-57D3-B14C-E5B3F76A5320; **Taxon:** scientificName: Absconditaperplexa (Walker, 1858); family: Lampyridae; **Location:** country: India; countryCode: Ind; stateProvince: West Bengal; locality: Naihati, North 24 Parganas; verbatimCoordinates: 22°52'48'' N, 88°25'48'' E; **Event:** samplingProtocol: Hand picking; eventDate: 27-September-18; **Record Level:** collectionCode: VUEC-0070; basisOfRecord: Preserved Specimen
- **Type status:** Other material. **Occurrence:** recordedBy: Srinjana Ghosh; sex: 2 males; occurrenceID: 905C7191-8EF5-5E6F-A4FE-4938E42B9C6F; **Taxon:** scientificName: Absconditaperplexa (Walker, 1858); family: Lampyridae; **Location:** country: India; countryCode: Ind; stateProvince: West Bengal; locality: Pailan, South 24 Parganas; verbatimCoordinates: 22°25'12'' N, 88°17'59'' E; **Event:** samplingProtocol: Hand picking; eventDate: 22-February-19; **Record Level:** collectionCode: VUEC-0076, VUEC-0077; basisOfRecord: Preserved Specimen
- **Type status:** Other material. **Occurrence:** recordedBy: Srinjana Ghosh; sex: 1 male, 3 females; occurrenceID: 55FF1428-40B5-5FDD-B7BB-01E815DCCA97; **Taxon:** scientificName: Absconditaperplexa (Walker, 1858); family: Lampyridae; **Location:** country: India; countryCode: Ind; stateProvince: Odisha; locality: Judia, Keonjhar; verbatimCoordinates: 21°37'48'' N, 86°34'12'' E; **Event:** samplingProtocol: Net sweeping; eventDate: 24- March-19; **Record Level:** collectionCode: VUEC-0060, VUEC-0061, VUEC-0062, VUEC-0063; basisOfRecord: Preserved Specimen

#### Diagnosis

Length 7–10 mm; Width 2.5–3.5 mm; *Abs.perplexa* is one of the three species of *Abscondita* (*Abs.chinensis*, *Abs.perplexa* and *Abs.terminalis*) present in India which is characterised by a pale dorsum with dark brown to black elytral apices (Fig. 4). PN, MN, MS and semi-transparent elytra pale brown, with the elytral apices black and occupying approximately 1/6 of its total length, head between eyes, labrum, antennae, palpi, apical part of tibiae and all tarsi dark brown, ventrites yellowish-orange, V6 and V7 occupying white LO. The species can be distinguished from other *Abscondita* species known from India by the following set of male characters: terminal abdominal tergites pale yellow (in *Abs.chinensis* and *Abs.terminalis*, abdominal tergites are dark brown), V5 bearing a medially notched and laterally broad dark patch along the posterior margin (in *Abs.chinensis*, the V5 is entirely dark). Male genitalia (Fig. 5): Aedeagus twice as long as wide (L:W = 0.5:0.25); ML longer than LL, gradually narrowing towards the apex, with the tip of ML projecting beyond the tips of LL (in *Abs.chinensis* and *Abs.terminalis*, both LL are longer than ML and the tip of ML is not projecting beyond the tips of LL), ML broader at base than LL; BP sclerotised, concave at its inner margin; LL subparallel-sided and fused with ML along most of the dorsal length, except in the separated apical 1/5 part; aedeagal sheath sternite terminated into two apically rounded lobes (in *Abs.chinensis*, the sheath sternite is terminated into two apically acute divergent lobes).

#### Biology

Adults of *Abs.perplexa* generally prefer open grassland area, but are also found on vegetation patches, near aquatic sites. Their oviposition sites include leaf litter or dry leaf beds. Larvae are nocturnal, glow weakly and were observed on plant debris and leaf litter in terrestrial habitats (Ballantyne et al. 2013).

#### Notes

This is a tentative identification, based solely on morphology.

### Asymmetricata ovalis

(Hope, 1831)

538085B0-BA1C-5610-884F-9E17F3B80D03

#### Materials

- **Type status:** Other material. **Occurrence:** recordedBy: Srinjana Ghos; sex: 2 males; occurrenceID: 9E8ED297-C7D3-5878-BCB7-3EEE003496B3; **Taxon:** scientificName: Asymmetricataovalis (Hope, 1831); family: Luciolinae; **Location:** country: India; countryCode: Ind; stateProvince: Odisha; locality: Kuldiha Wildlife Sanctuary, Balasore; verbatimCoordinates: 21°25'12'' N, 86°43'48'' E; **Event:** samplingProtocol: Net sweeping; **Record Level:** collectionCode: VUEC-0054, VUEC-0055; basisOfRecord: Preserved Specimen

#### Diagnosis

Length 9–9.5 mm; Width 3.2–3.5 mm. Ventral thorax yellow, V3-V5 dark brown, LO white, legs yellow with the apical part of tibiae and all tarsi dark brown (Fig. 6). *Asy.ovalis* can be distinguished from other species of the genus by the following set of male characters: elytra dark brown to black with the sutural and lateral margins pale yellow (*Asy.bicoloripes* is with pale yellowish-brown dorsum, elytra pale brown bearing dark brown basal and apical parts in *Asy.humeralis*); PN brownish-orange (in *Asy.humeralis*, there are paired median dark spots on pronotum); V6 and V7 having white LO with the LO in V7 bipartite (whereas LO is entire in *Asy.circumdata*); base of PN bisinuate; ASD < ASW; apex of MPP narrowly rounded. Male genitalia (Fig. 7): Aedeagus longer than wide (L/W =1.8); LL are of equal length and slightly shorter than ML (length of LL/length of ML = 0.6), asymmetrical, inner lateral margin of LL diverging from ML for about 4/5 of their length, outer lateral margin sinuate, apex of right LL out-turned, LL apex width greater than ML apex width, ML pointed and little produced beyond LL, BP hooded with its margin facing ML and LL, concave and covering the basal parts of ML and LL; aedeagal sheath L/W= 3.75, posteriorly symmetrical and subparallel-sided, posterior margin of sheath sternite broad and slightly asymmetrically emarginated, anterior part of sternite apically rounded; lateral arms of sheath tergite extended narrowly to the anterior sides of sheath sternite.

#### Biology

Adult males of *Asy.ovalis* were found flying at a height of around 5 metres, often reaching the mid- to upper canopy level in forest habitat. Some of these were collected when they came down by sweep net and identified in the laboratory. Females and larvae are terrestrial (Ballantyne and Lambkin 2009, Ballantyne et al. 2019).

### Pteroptyx malaccae

(Gorham, 1880)

32CE3E02-7C99-5856-A61E-1B43E57FD303

#### Materials

- **Type status:** Other material. **Occurrence:** recordedBy: Srinjana Ghosh; sex: 1 male; occurrenceID: 04F5F577-6780-5BBF-B254-AD5ACE09CCF3; **Taxon:** scientificName: Pteroptyxmalaccae (Gorham, 1880); family: Lampyridae; **Location:** country: India; countryCode: Ind; locality: near Sajnekhali Bird Sanctuary, Sundarban Biosphere Reserve, South 24 Parganas; verbatimCoordinates: 22°7'12'' N, 88°46'49'' E; **Event:** samplingProtocol: Hand picking; **Record Level:** collectionCode: VUEC-0001; basisOfRecord: Preserved Specimen
- **Type status:** Other material. **Occurrence:** recordedBy: Srinjana Ghosh; sex: 1 male; occurrenceID: 5EBAF084-18C2-5437-A4A8-B7855CA5CF0C; **Taxon:** scientificName: Pteroptyxmalaccae (Gorham, 1880); family: Lampyridae; **Location:** country: India; countryCode: Ind; locality: Dhainchi island, South 24 Parganas; verbatimCoordinates: 21°42'05'' N, 88°26'00'' E; **Event:** samplingProtocol: Hand picking; **Record Level:** collectionCode: VUEC-0035; basisOfRecord: Preserved Specimen
- **Type status:** Other material. **Occurrence:** recordedBy: Srinjana Ghosh; sex: 1 male; occurrenceID: 6C7729A3-6B46-53FB-BD57-3E18E0899940; **Taxon:** scientificName: Pteroptyxmalaccae (Gorham, 1880); family: Lampyridae; **Location:** country: India; countryCode: Ind; locality: Bonnie Island, South 24 Parganas; verbatimCoordinates: 21°49'50'' N, 88°37'26'' E; **Event:** samplingProtocol: Hand picking; **Record Level:** collectionCode: VUEC-0081; basisOfRecord: Preserved Specimen

#### Diagnosis

Length 5–6.5 mm; Width 1.5–1.8 mm; PN, MN and MS brownish-yellow, thorax ventrally yellowish-orange, elytra bright brownish-yellow with its apices black, head between eyes, antennae and palpi dark brown, legs brownish-yellow with the tibae and tarsi brown, terminal abdominal tergites yellow, ventrites brownish-yellow, tip of PLP brown, V6 and V7 having white LO with the LO in V7 bipartite (Fig. 8). *Pt.malaccae* can be distinguished from other species of the genus by the following set of male characters: apex of FS1 strongly produced laterally; elytra almost parallel-sided with its apices deflexed and lacking depressions (*Pt.gelasina* possesses depressions at the elytral apices); elytral apices twice as long as wide (*Pt.truncata*, in contrast, bears comparatively shortened elytral apices); hind-tibae expanded; posterior margin of T8 without lobes; V7 with a broad and deep semicircular emargination along its posterior margin separating the MPP and PLP, with the PLP slenderly produced (PLP is comparatively narrower in *Pt.gelasina* and, in *Pt.maipo*, *Pt.sulawesiensis* and *Pt.valida*, the PLP of V7 are scarcely produced and broadly rounded). Male genitalia (Fig. 9): Aedeagus slender, elongated, symmetrical, aedeagus b/a 0.9, LL are of equal length, slightly shorter than ML, separated by half of their dorsal length, lack lateral hairy appendages and slender leaf-like projections along their outer ventral margins and inner margins respectively, ML symmetrical, narrow and tube-like, not bearing paired lateral teeth and without ventral inclination, BP bluntly pointed, lightly sclerotised; aedeagal sheath symmetrical, apically rounded, aedeagal sheath sternite relatively narrower at the anterior half, widest at the middle and tapers almost symmetrically towards an entire narrow apex, paraprocts bulbous, L/W = 3/1.

#### Biology

Adults of *Pt.malaccae* were found in mangrove patches and synchronous flashing was observed on different display plants, like *Avicenniaalba* Blume and *Avicenniaofficinalis* L. The fireflies form small aggregations of 6 to 10 individuals and the males were found flashing during flight. The specimens were observed flying at an average height of 7 to 8 metres and some of them were collected when they came down by sweep net. The collected specimens were identified in the laboratory.

### Sclerotia aquatilis

(Thancharoen, 2007)

7CF71C64-1185-5EBE-B5DC-1896CDF7300E

#### Materials

- **Type status:** Other material. **Occurrence:** recordedBy: Srinjana Ghosh; sex: 3 males; occurrenceID: E4AA985E-C1E3-5AA1-AE21-F90D309A65D0; **Taxon:** scientificName: Sclerotiaaquatilis (Thancharoen, 2007); family: Lampyridae; **Location:** country: India; countryCode: Ind; stateProvince: West Benga; locality: Kulpi, South 24 Parganas; verbatimCoordinates: 22°4'48'' N, 88°17'60'' E; **Event:** samplingProtocol: Net sweeping; eventDate: 10-September-17; **Record Level:** collectionCode: VUEC-0025, VUEC-0026, VUEC-0027; basisOfRecord: Preserved Specimen
- **Type status:** Other material. **Occurrence:** recordedBy: Srinjana Ghosh; sex: 1 male; occurrenceID: CDFE2902-8558-5D26-82DF-A912DDAAB01A; **Taxon:** scientificName: Sclerotiaaquatilis (Thancharoen, 2007); family: Lampyridae; **Location:** country: India; countryCode: Ind; stateProvince: Odisha; locality: near Chandipore coast, Balasore; verbatimCoordinates: 21°29'36'' N, 86°55'19''E; **Event:** samplingProtocol: Net sweeping; eventDate: 8-May-18; **Record Level:** collectionCode: VUEC-0025, VUEC-0026, VUEC-0027; basisOfRecord: Preserved Specimen
- **Type status:** Other material. **Occurrence:** recordedBy: Srinjana Ghosh; sex: 2 males; occurrenceID: 2AD51D56-287E-5592-B332-59E3E88F9371; **Taxon:** scientificName: Sclerotiaaquatilis (Thancharoen, 2007); family: Lampyridae; **Location:** country: India; countryCode: Ind; stateProvince: West Bengal; locality: Egra, East Midnapore; verbatimCoordinates: 21°53' 56'' N, 87°31'48'' E; **Event:** samplingProtocol: Hand picking; eventDate: 11-August-18; **Record Level:** collectionCode: VUEC-0063, VUEC-0064; basisOfRecord: Preserved Specimen
- **Type status:** Other material. **Occurrence:** recordedBy: Srinjana Ghosh; sex: 4 males, 3 females; occurrenceID: 8563A1F5-DE6F-5516-8B68-467F2BE5DB2D; **Taxon:** scientificName: Sclerotiaaquatilis (Thancharoen, 2007); family: Lampyridae; **Location:** country: India; countryCode: Ind; stateProvince: West Bengal; locality: near Sajnekhali Bird Sanctuary, Sundarban Biosphere Reserve, South 24 Parganas; verbatimCoordinates: 22°7'12''N, 88°46'49'' E; **Event:** samplingProtocol: Net sweeping; eventDate: 14-July-19; **Record Level:** collectionCode: VUEC-0096, VUEC-0097, VUEC-0098, VUEC-0099, VUEC-0100, VUEC-0101, VUEC-0102; basisOfRecord: Preserved Specimen

#### Diagnosis

Length 9–11 mm; Width 3.5–4 mm; PN, MN and MS pale brownish-yellow, elytra dark brown with the sutural and lateral margins bright yellow, ventrites pale brown, legs pale brown with the apical parts of tibae and entire tarsi dark brown (Fig. 10). *Scl.aquatilis* can be distinguished from *Scl.substriata* (the other known species of the genus from India) by the following set of male characters: V4 and V5 having dark brown patches along the posterior margin with the dark patch of V5 occupying approximately 2/3 of the part posteriorly (in case of *Scl.substriata*, whole part of V4 and V5 dark brown in colour); LO occupies the entire V6 and V7, except for a small median emargination at the anterior part of V7 (in case of *Scl.substriata*, the anterior median emargination of the LO in V7 is of comparatively greater depth). Male genitalia (Fig. 11): Aedeagus L/W 2.1, aedeagal sheath sternite having an oblique bar and a small median posterior emargination, anteromedian margin of sheath tergite emarginated (in case of *Scl.substriata*, the transverse oblique ridge in posterior half of sheath sternite is not present, elongate pointed projection of the right side of the posterior margin of the sheath sternite is not found and there is rounded prolongation of the median anterior margin of the aedeagal sheath tergite); Sclerites (Fig. 11a, b) - distal end of triangular-shaped ventral sclerite is rounded in shape, its margin is not sclerotised, anterolateral corners acute; right sclerite possesses three projections to the right, of those, the posterior one apically acute, median one is dorsolaterally inclined with the apical end slender and rounded, the third one is the longest, arises in an emargination between the other two, ventrolaterally inclined with rounded hollow apex; left sclerite also possesses three arms, all of which are apically rounded with the anterior one expanded.

#### Biology

Males of *Scl.aquatilis* were found perching on macrophyte surfaces at the onset of darkness. Their average flight height ranged between 2 to 5 metres. Females are macropterous. Metapneustic larvae are exclusively aquatic in nature and the later instars lack gills, while in initial instars, lateral bristles function as gills (Fu and Ballantyne 2009). The 1^st^ – 2^nd^ instar larvae obtain dissolved oxygen through tracheal gills, whereas the 3^rd^ – 6^th^ instar back-swimmer larvae, without tracheal gills, swim just below the water surface and expose their abdominal segments to air to breath by their terminal spiracles (Fu et al. 2005, Zheng et al. 2008). Adult individuals were observed near aquatic sites.

### Sclerotia substriata

(Gorham, 1880)

0B272C84-8696-5DC5-84A8-41A03832667F

#### Materials

- **Type status:** Other material. **Occurrence:** recordedBy: Santu Paria; sex: 1 male; occurrenceID: E12FC091-3CE3-5559-AC50-16E014DDE237; **Taxon:** scientificName: Sclerotiasubstriata (Gorham, 1880); family: Lampyridae; **Location:** country: India; countryCode: Ind; stateProvince: West Bengal; locality: East Midnapore; locality: Egra; verbatimCoordinates: 21°53'60''N, 87°31'48'' E; **Event:** samplingProtocol: Hand picking; eventDate: 11-August-18; **Record Level:** collectionCode: VUEC-0065; basisOfRecord: Preserved Specimen
- **Type status:** Other material. **Occurrence:** recordedBy: Srinjana Ghosh; sex: 3 males; occurrenceID: FA0BE07B-3E4F-5C41-A991-283E8E5A2060; **Taxon:** scientificName: Sclerotiasubstriata (Gorham, 1880); family: Lampyridae; **Location:** country: India; countryCode: Ind; stateProvince: West Bengal; locality: Aamtala, South 24 Parganas; verbatimCoordinates: 22°21'59'' N, 88°16'37''; **Event:** samplingProtocol: Net sweeping; eventDate: 14-September-19; **Record Level:** collectionCode: VUEC-0103, VUEC-0104, VUEC-0105; basisOfRecord: Preserved Specimen

#### Diagnosis

Length 8.4 mm; Width 3.8 mm; PN, MN and MS yellowish-orange, elytra light greyish-brown with its apices black tipped and lateral and sutural margins bright yellow, legs yellow with the apical parts of tibae and entire tarsi brownish-black (Fig. 12). *Scl.substriata* can be distinguished from *Scl.aquatilis* (the other known species of the genus from India) by the following set of male characters: V3, V4, V5 brownish-black (in case of *Scl.aquatilis*, V4 and V5 having dark brown patches along the posterior margin with the dark patch of V5 occupying approximately 2/3 of the part posteriorly), LO occupies the entire V6 and V7, with the V7 having a moderately deep (approximately 1/3 of V7 length) median emargination at its anterior margin (in case of *Scl.aquatilis*, a small median emargination at the anterior part of V7). Male genitalia (Fig. 13): Aedeagus L/W 2.1, aedeagal sheath sternite bears no oblique median ridge at posterior part and anteromedian margin of sheath tergite with rounded prolongation (in case of *Scl.aquatilis*, aedeagal sheath sternite having an oblique bar and a small median posterior emargination, anteromedian margin of sheath tergite emarginated); Sclerites (Fig. 13a, b) - ventral sclerite almost circular with asymmetrically thickened ventral surface, a curved band lying in the middle; right sclerite bears anterior ventral projection which is hollow and apically rounded; at its side, there lies a curved anteriorly pointed projection, minute lateral projections become fused and lie behind and beneath both of ventral and right sclerites as a broad piece with irregular lateral margins; left sclerite has three lateral slender and pointed projections, the inner arm of median and posterior projections are curved and apically pointed.

#### Biology

Males of *Scl.substriata* were found in flight up to canopy heights of 6 to 7 metres and over macrophyte surfaces during nocturnal hours. On shining of a bright light, the individuals stop flying and drop. Females are macropterous. Metapneustic larvae are exclusively aquatic in nature and the later instars lack tracheal gills, while in initial instars, lateral bristles function as gills (Fu and Ballantyne 2009). The 1^st^ – 2^nd^ instar larvae obtain dissolved oxygen through tracheal gills, whereas the 3^rd^ – 6^th^ instar back-swimmer larvae, without tracheal gills, swim just below the water surface and expose their abdominal segments to air to breath by their terminal spiracles (Fu et al. 2005, Zheng et al. 2008). Adult individuals were found near aquatic sites.

## Discussion

The present faunistic report, based on the occurrence and distribution of fireflies in the two eastern States of India, reflects that a number of firefly species, having wider distribution throughout SE Asia, also inhabit diverse habitat types (grassland, forest, marshy area, roadside vegetation patches etc., as mentioned in the description of the study sites), which include *Abs.chinensis*, *Abs.terminalis*, *Pt.malaccae* and *Asy.ovalis*. These species were reported earlier from a number of countries in this geographical subregion of South Eastern and Southern Asia. On the contrary, species like *Abs.perplexa* (Cambodia, India, Sri Lanka), *Asy.humeralis* (India, Indonesia, Sri Lanka), *Scl.aquatilis* (India, Laos, Thailand, Vietnam) and *Scl.substriata* (India, Myanmar, Sri Lanka) (Gorham 1880, Ballantyne et al. 2013, Ballantyne et al. 2016, Ballantyne et al. 2019, Seri and Rahman 2021) have demonstrated scattered distribution which is revealed by their presence in fewer countries of this global subregion.

McDermott (1966) listed 279 species under the subgenus Luciola and he had (McDermott 1964) previously indicated the necessity to subdivide some of them. Based on McDermott’s arrangement, species identification and taxonomy of *Luciola* was imprecise and leading to many erroneous grouping and taxonomic instability (Ballantyne et al. 2019, Jusoh et al. 2021). To provide an appropriate taxonomic framework for the *Luciola* fauna of the SE Asia and the Australopacific Region and also to define what *Luciola* s. str. could contain, Ballantyne et al. (2019) and Jusoh et al. (2021) addressed and defined *Luciola* s. str. including 17 species previously listed under the subgenus Luciola in McDermott (1966). This definition allowed the erection of many new genera involving transfers of species from Luciola (Luciola) sensu McDermott (1966). In their work, Ballantyne et al. (2019) recommended for transfer of seven species (including *L.costata* Pic) to the genus *Curtos*, assigned 33 species (including two synonyms) to *Luciola* s. lato and treated 35 species as species incertae sedis.

Most recently, the status of the genus *Luciola* s. str. has been updated with erection of a new genus *Nipponoluciola* Ballantyne, Kawashima, Jusoh & Suzuki (Ballantyne et al. 2022). The new genus has been erected for two species of Japanese fireflies *Nipponoluciolacruciata* (Motschulsky) and *Nipponoluciolaowadai* (Satô & Kimura) possessing aquatic larvae. They have also suggested for placements of many of remaining species listed under *Luciola* in Ballantyne et al. (2019) and mentioned the problems in proper taxonomic arrangement of a few of these species, like misidentification of specimens, loss of holotypes, mislabelling, inadequacy of original descriptions, degradation of specimens in museums as an effect of longer course of preservation, larger cost of obtaining support from European museums, where most of the types are located and the difficulties imposed during Covid situation at global level from 2020 (Ballantyne et al. 2022).

The Indian checklist (Table 1) presented in this work, reflecting only about 8% (33 out of 400) of the World Luciolinae firefly fauna, clearly shows the need for more taxonomic studies on this group to be carried out in the country. The listed species of the country exhibit about 33% endemism (11 out of 33 species endemic to India). The highest number of species was recorded from the State of West Bengal (11), followed by Tamilnadu (seven), Odisha (five), Karnataka and Maharashtra (four each), Assam (three) and Andaman and Nicobar Islands, Goa and Kerala (two each). Unfortunately, only a single species has been reported from Bihar, Madhya Pradesh, Meghalaya, Pondicherry and Sikkim. Out of the total species recorded in India, *Abs.chinensis* (from five States), *Abs.perplexa* (from four States), *Asy.humeralis* and *Asy.ovalis* (each from three States), *Scl.aquatilis* and *Scl.substriata* (each from three States), *Abs.terminalis* and *Pt.malaccae* (each from two States) could be considered as the prevalent firefly species of India. On the contrary, *Py.insularis* has been reported exclusively from Andaman and Nicobar Islands and *Asy.circumdata* from the Meghalaya State indicating a localised distribution. The distribution pattern of Indian species indicates that *Abs.chinensis*, *Abs.perplexa*, *Abs.terminalis*, *Asy.humeralis*, *Asy.ovalis* and *Scl.aquatilis* are the better studied and widely distributed species in India. However, for the rest of the species, either they are reported from only one/two States or their precise distribution in India is not known.

*Abs.anceyi*, *Abs.pallescens* and *Triangularafrontoflava*, which were previously reported from India in Ghosh et al. (2021), are not included in this article because the identity of *Abs.anceyi* could not be confirmed due to lack of male specimens, *Abs.pallescens* is incorrectly reported from India and *Tr.frontoflava* has been misidentified in Ghosh et al. (2021). The checklist and distributional record of species presented herein has been prepared only on the basis of published articles and some records which were presented earlier in Ghosh et al. (2021) are not included here as those were incorrectly reported or mistakenly incorporated from unpublished documents.

In the present study, the recorded firefly species show diversified habitat preferences. Of all the study sites, mangroves of Sundarban Biosphere Reserve support the highest diversity of Luciolinae species, providing necessary environmental resources like wetland association, vegetation compositions and shelter for larvae and adults. During our field study, we found that dependence on display plants and association with wetlands are prominent factors influencing abundance and distribution of firefly species.

## Supplementary Material

XML Treatment for Abscondita chinensis

XML Treatment for Abscondita perplexa

XML Treatment for Asymmetricata ovalis

XML Treatment for Pteroptyx malaccae

XML Treatment for Sclerotia aquatilis

XML Treatment for Sclerotia substriata

## Figures and Tables

**Figure 2a.**
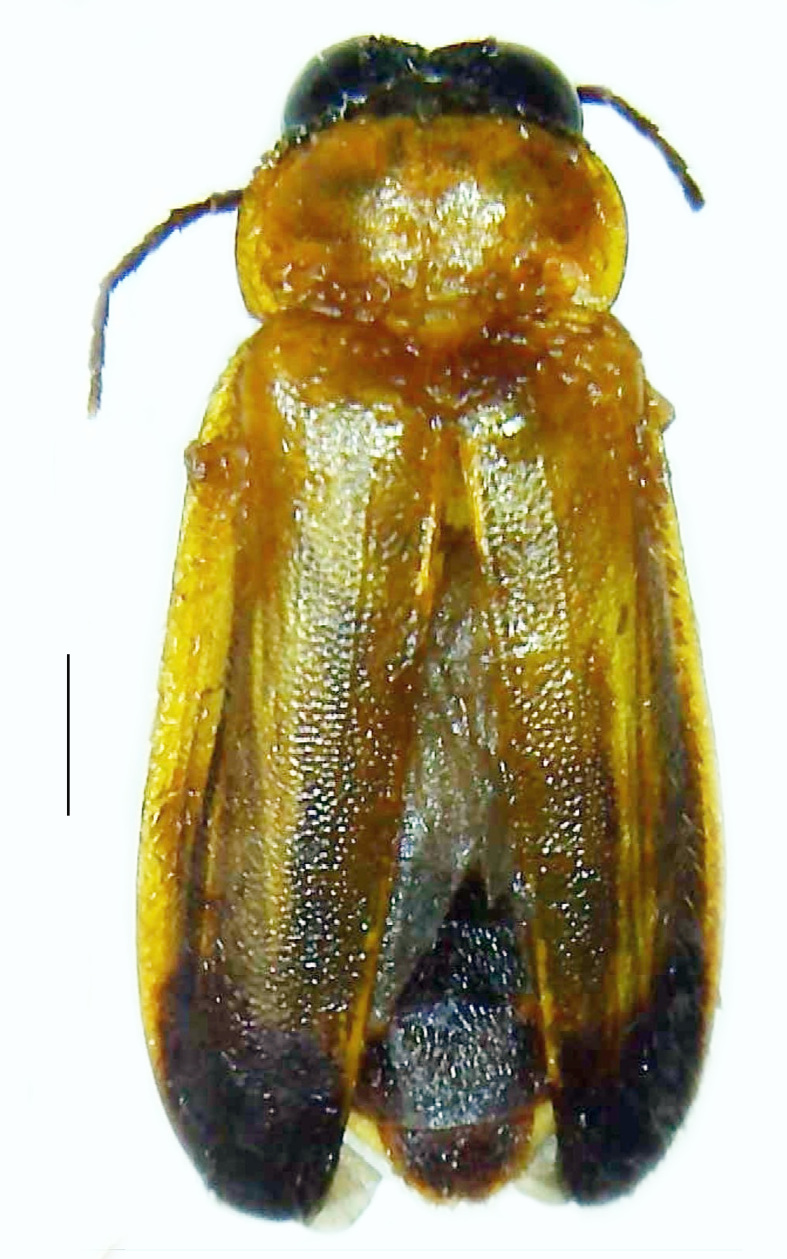
Male dorsal habitus. Scale bar: 1 mm;

**Figure 2b.**
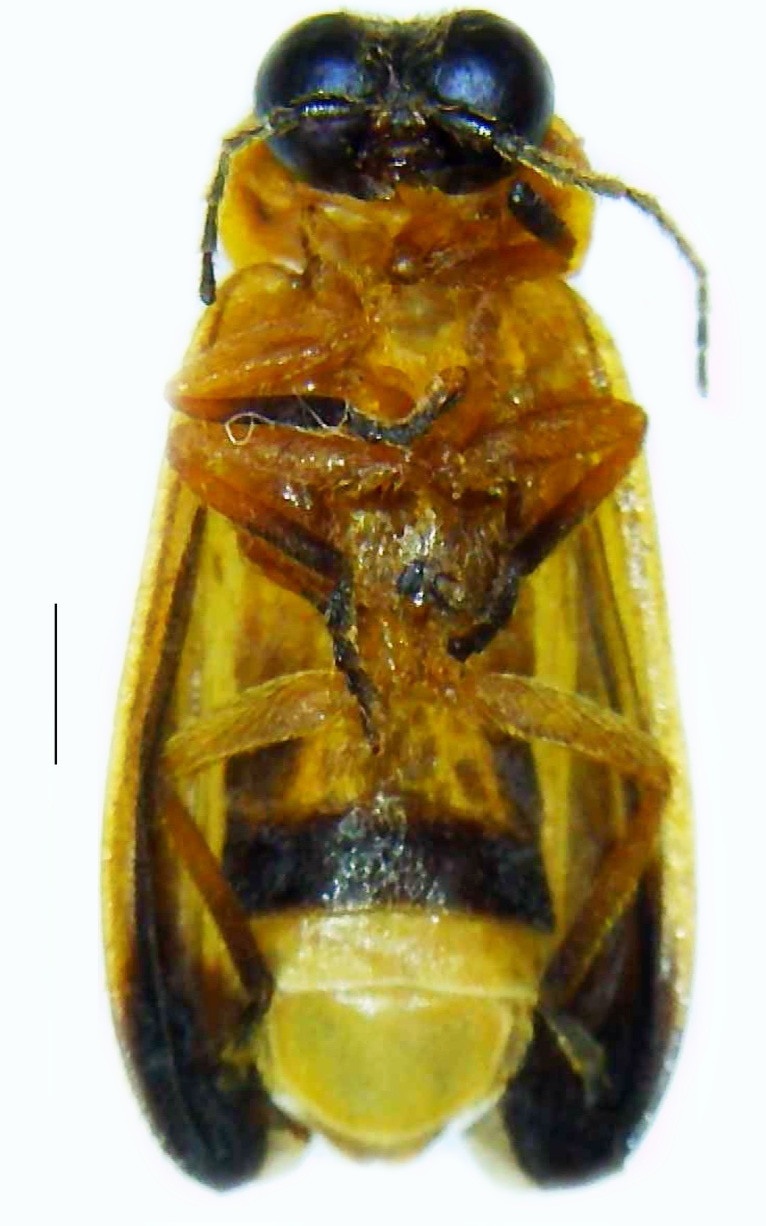
Male ventral habitus. Scale bar: 1 mm;

**Figure 2c.**
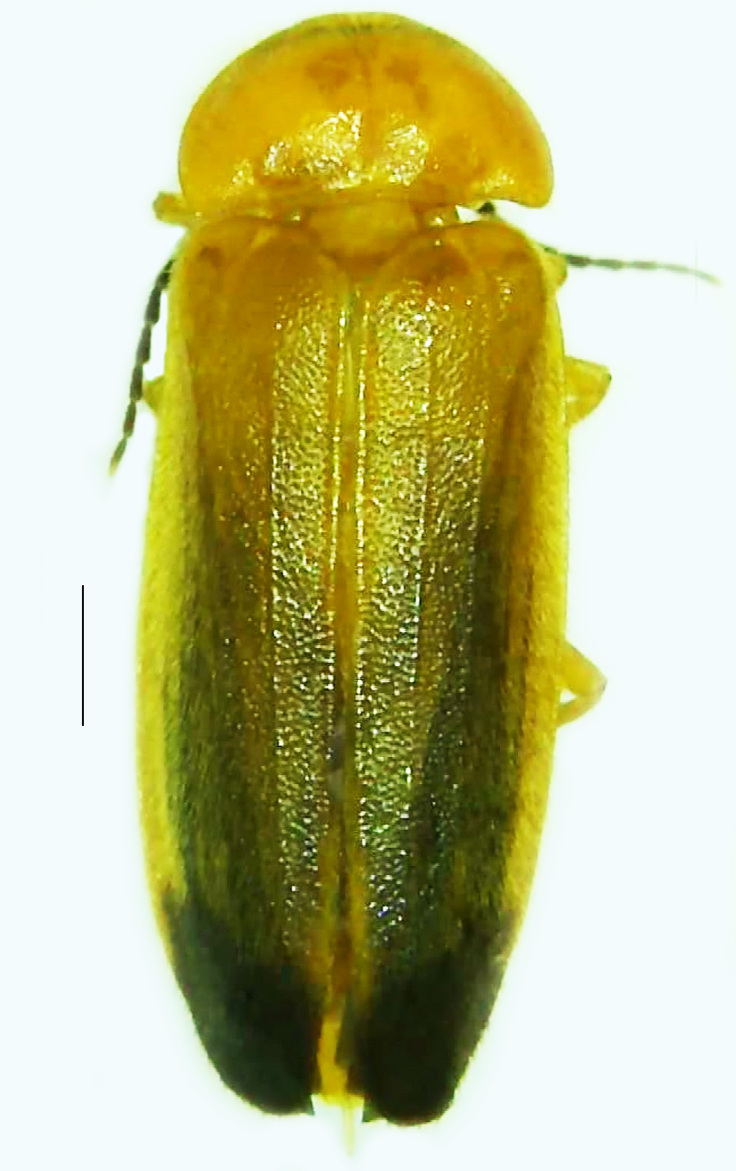
Female dorsal habitus. Scale bar: 1 mm;

**Figure 2d.**
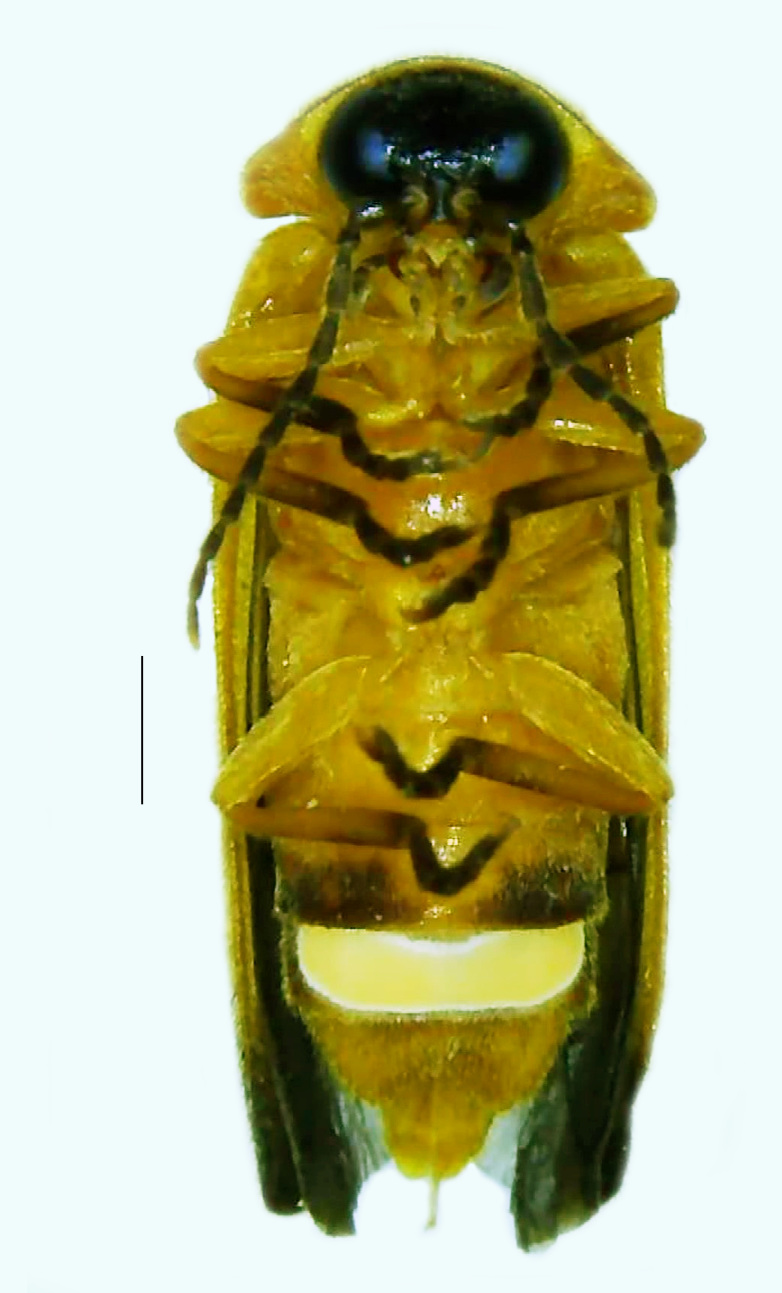
Female ventral habitus. Scale bar: 1 mm.

**Figure 3a.**
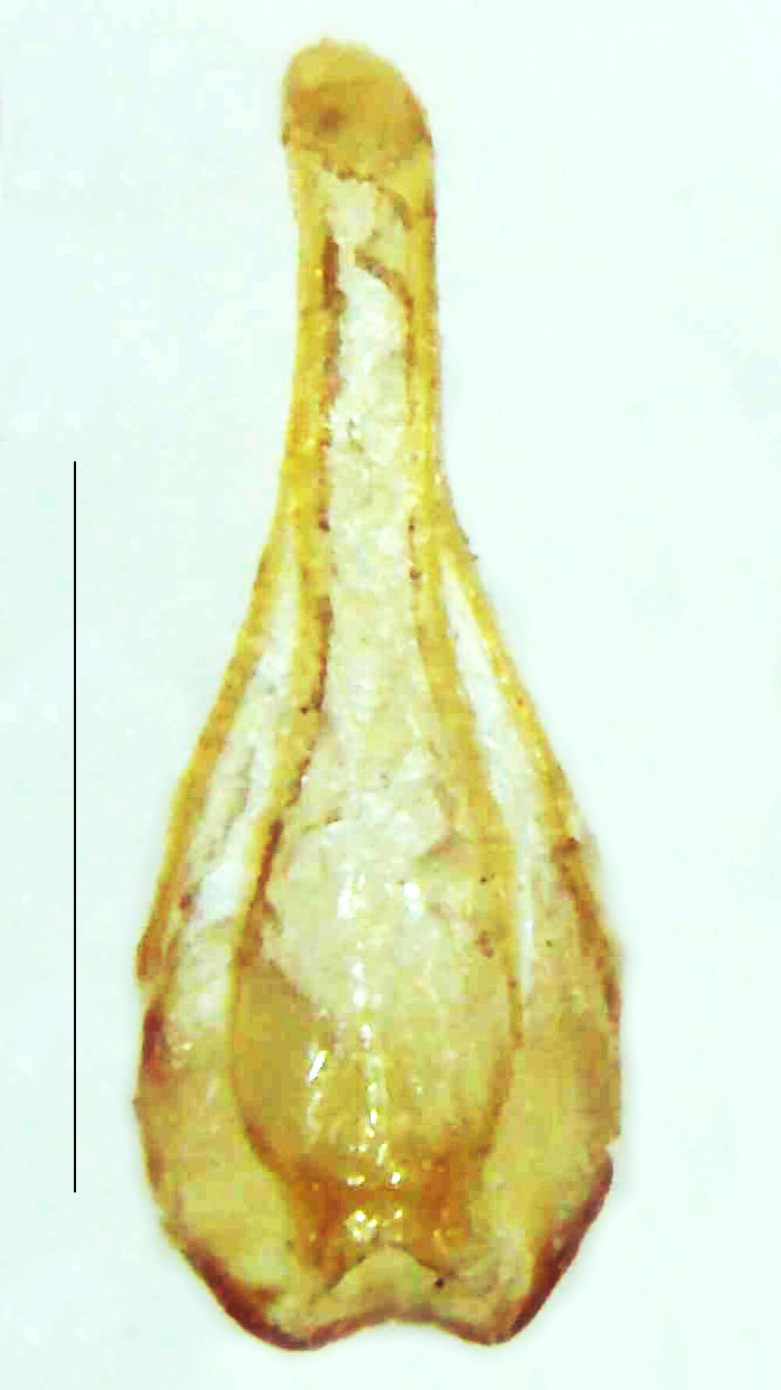
Aedeagal sheath dorsal. Scale bar: 0.5 mm;

**Figure 3b.**
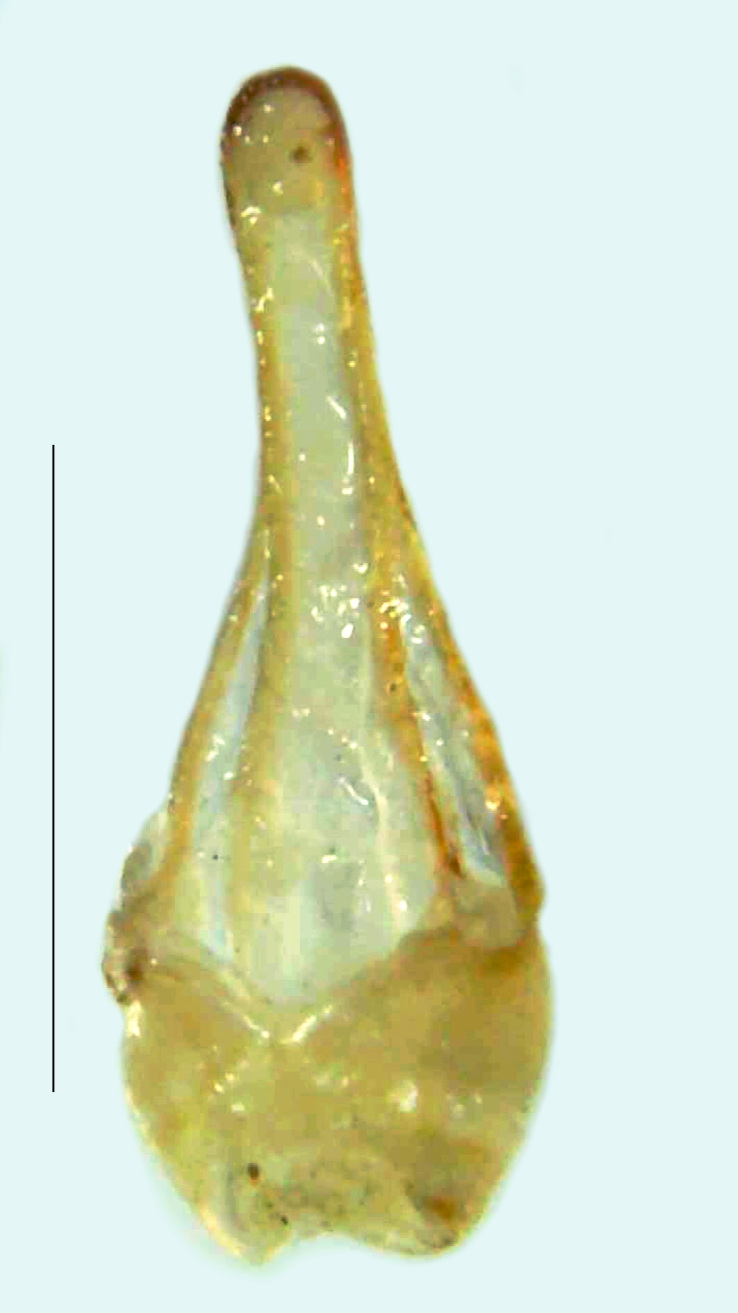
Aedeagal sheath ventral. Scale bar: 0.5 mm;

**Figure 3c.**
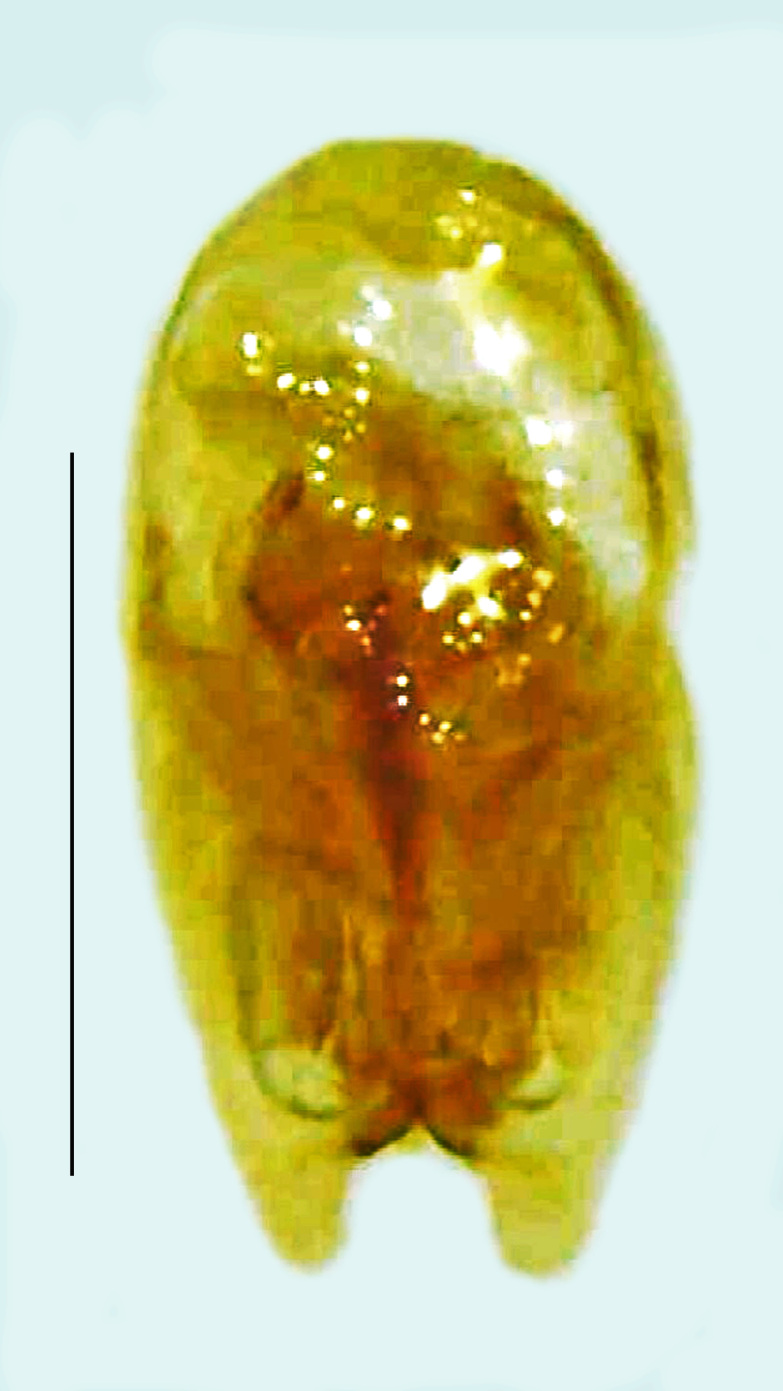
Aedeagus dorsal. Scale bar: 0.5 mm;

**Figure 3d.**
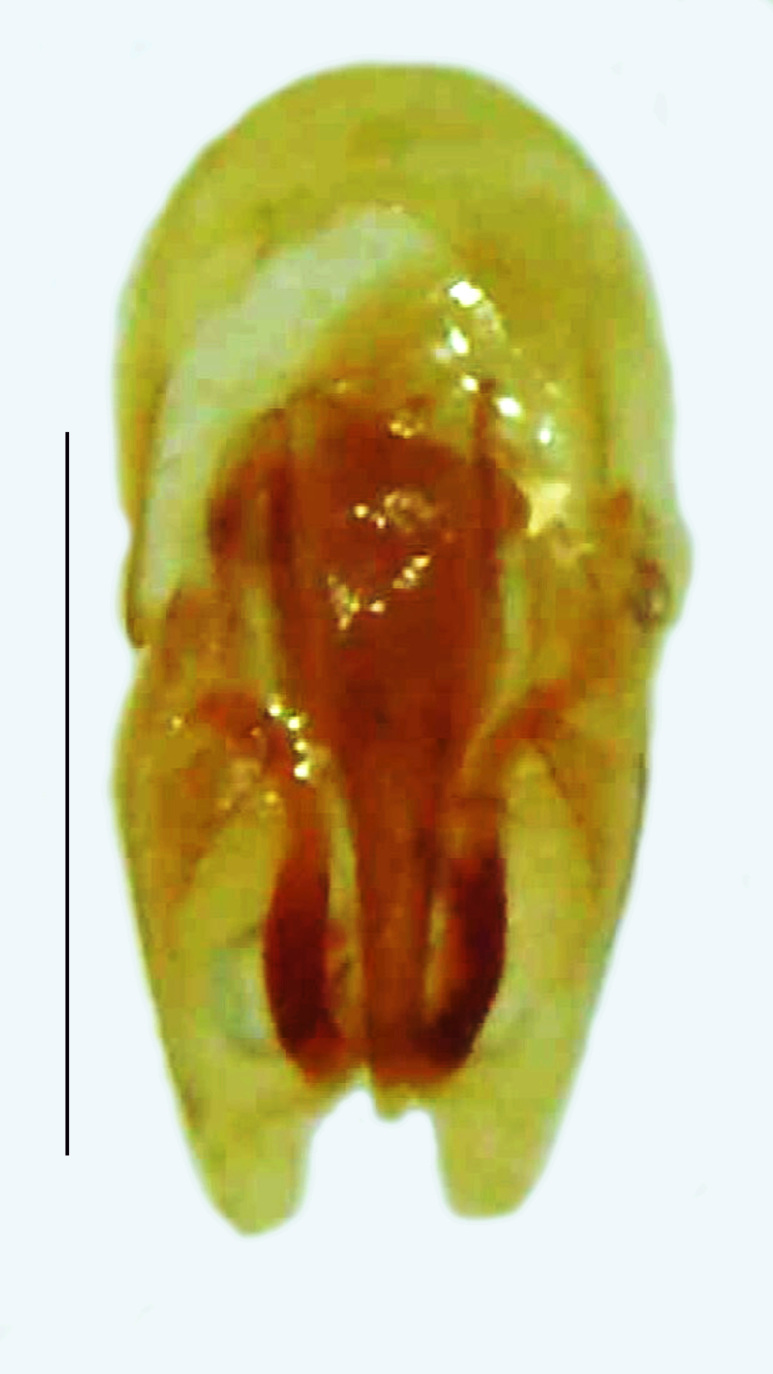
Aedeagus ventral. Scale bar: 0.5 mm.

**Figure 4a.**
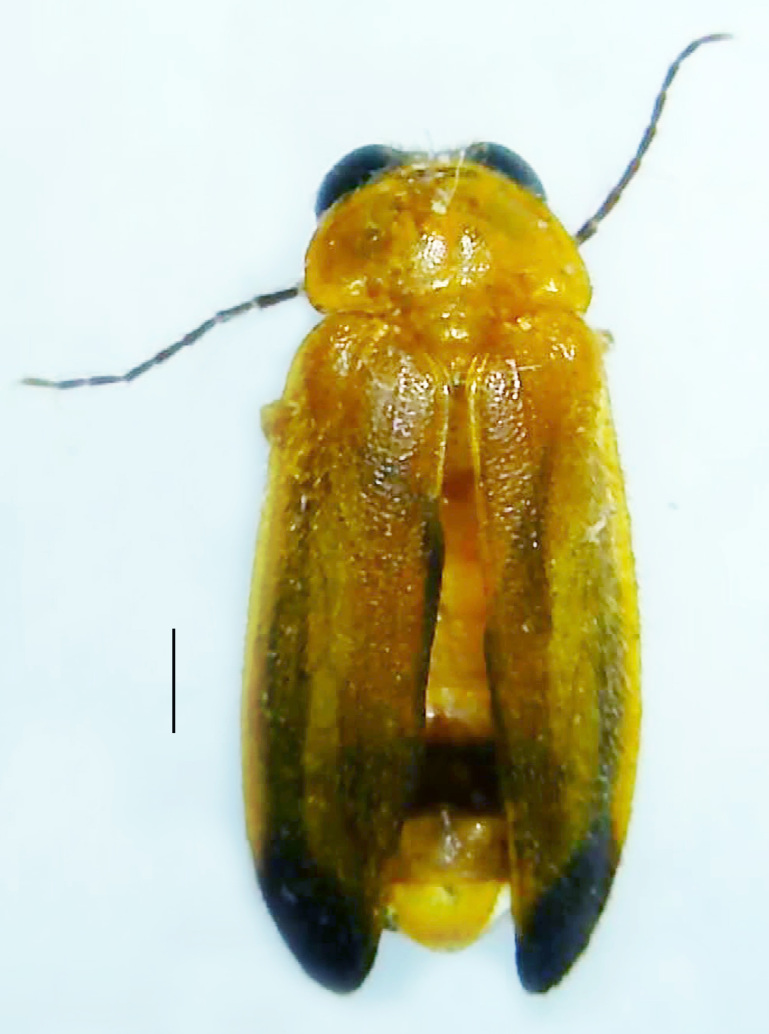
Male dorsal habitus. Scale bar: 1 mm;

**Figure 4b.**
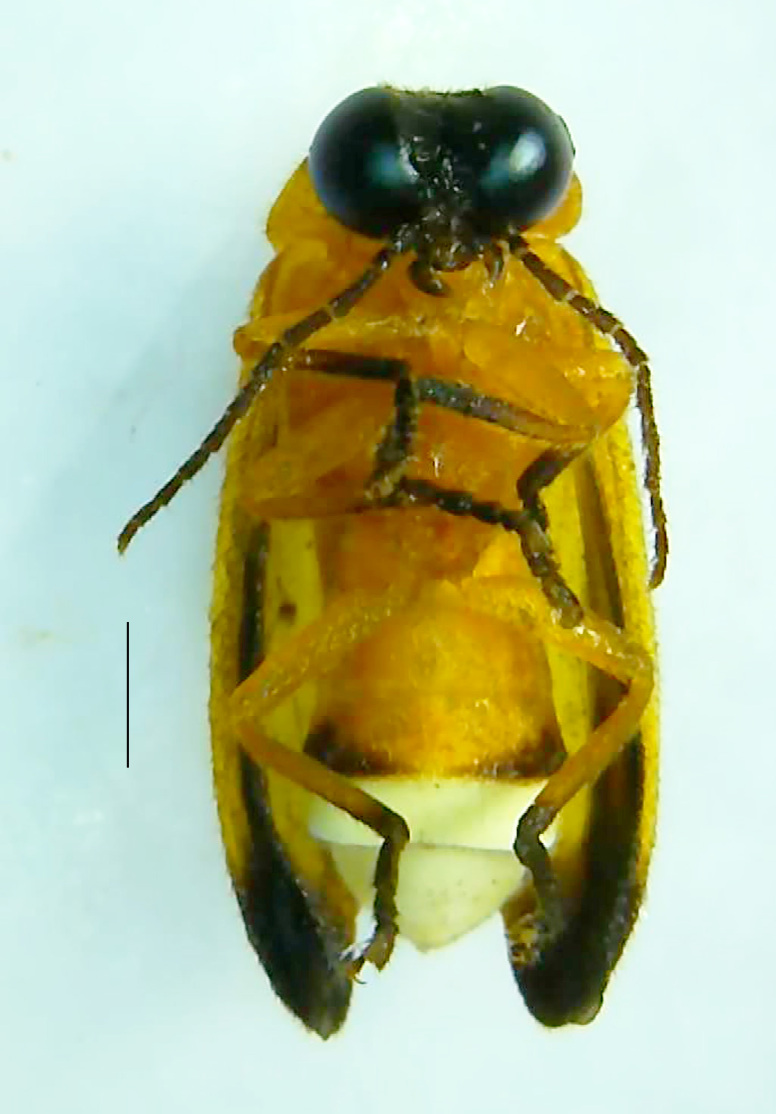
Male ventral habitus. Scale bar: 1 mm;

**Figure 4c.**
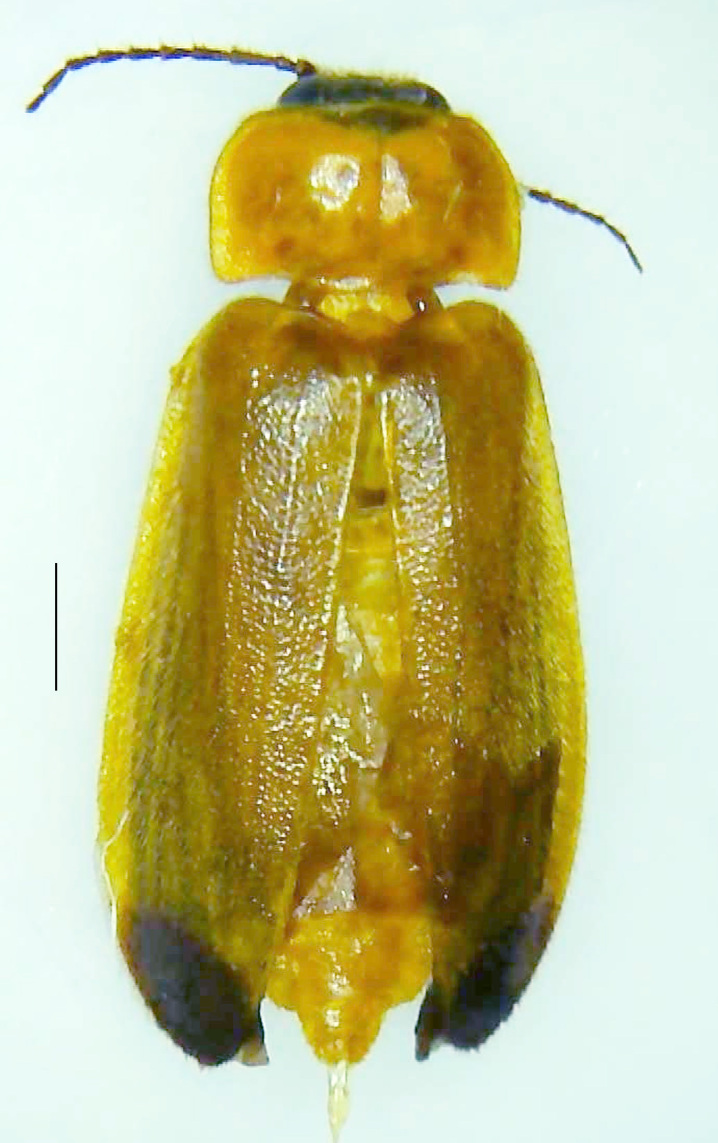
Female dorsal habitus. Scale bar: 1 mm;

**Figure 4d.**
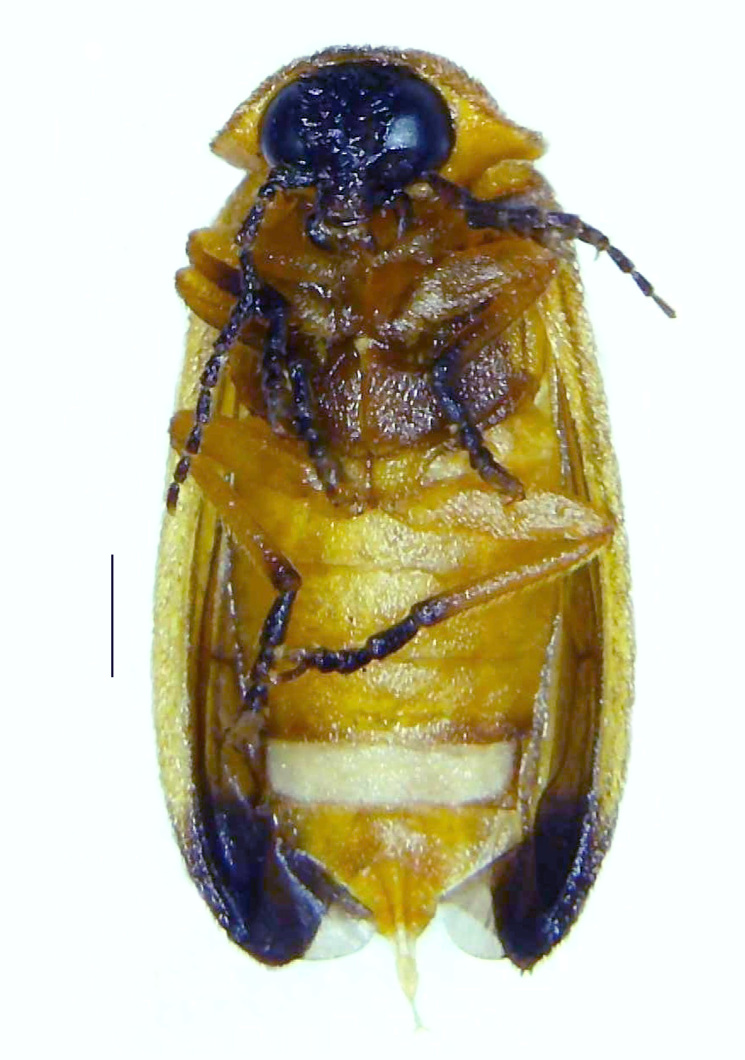
Female ventral habitus. Scale bar: 1 mm.

**Figure 5a.**
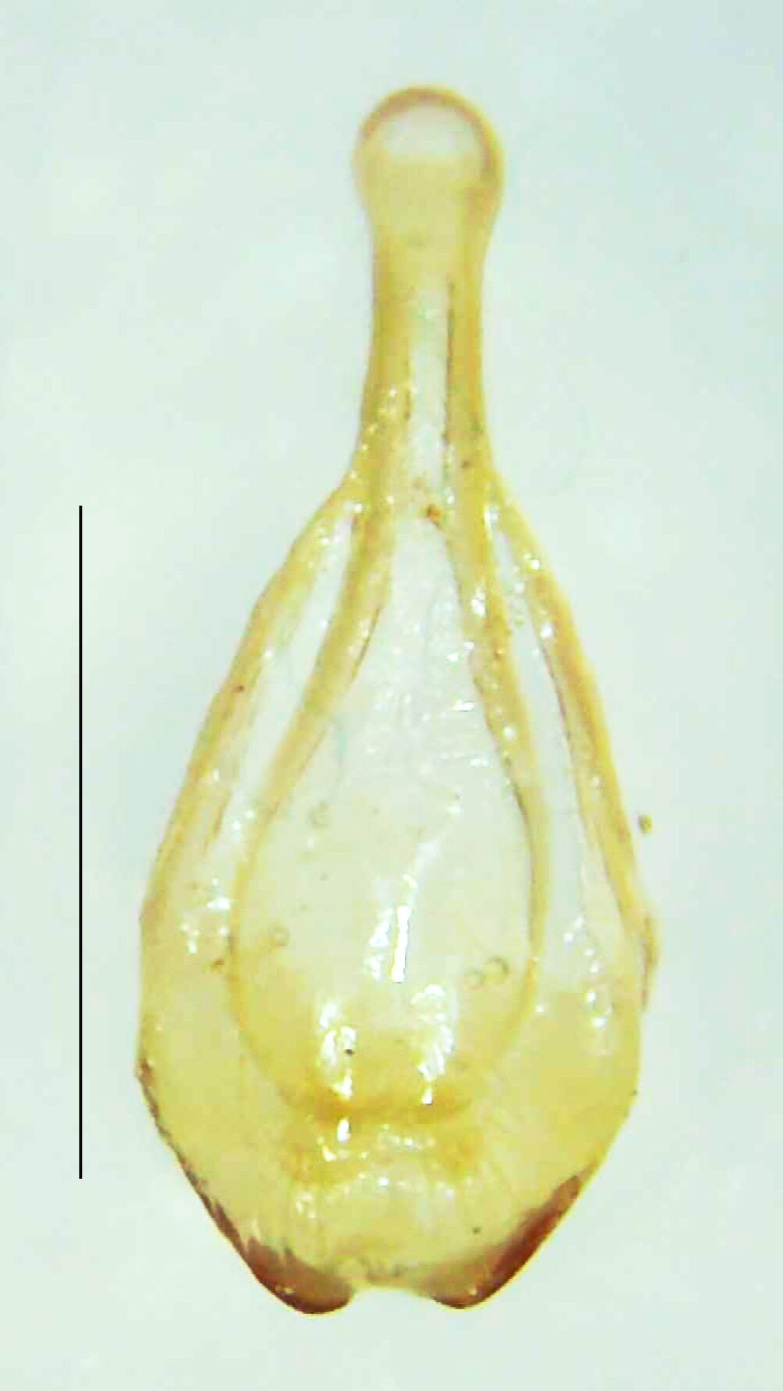
Aedeagal sheath dorsal. Scale bar: 0.5 mm;

**Figure 5b.**
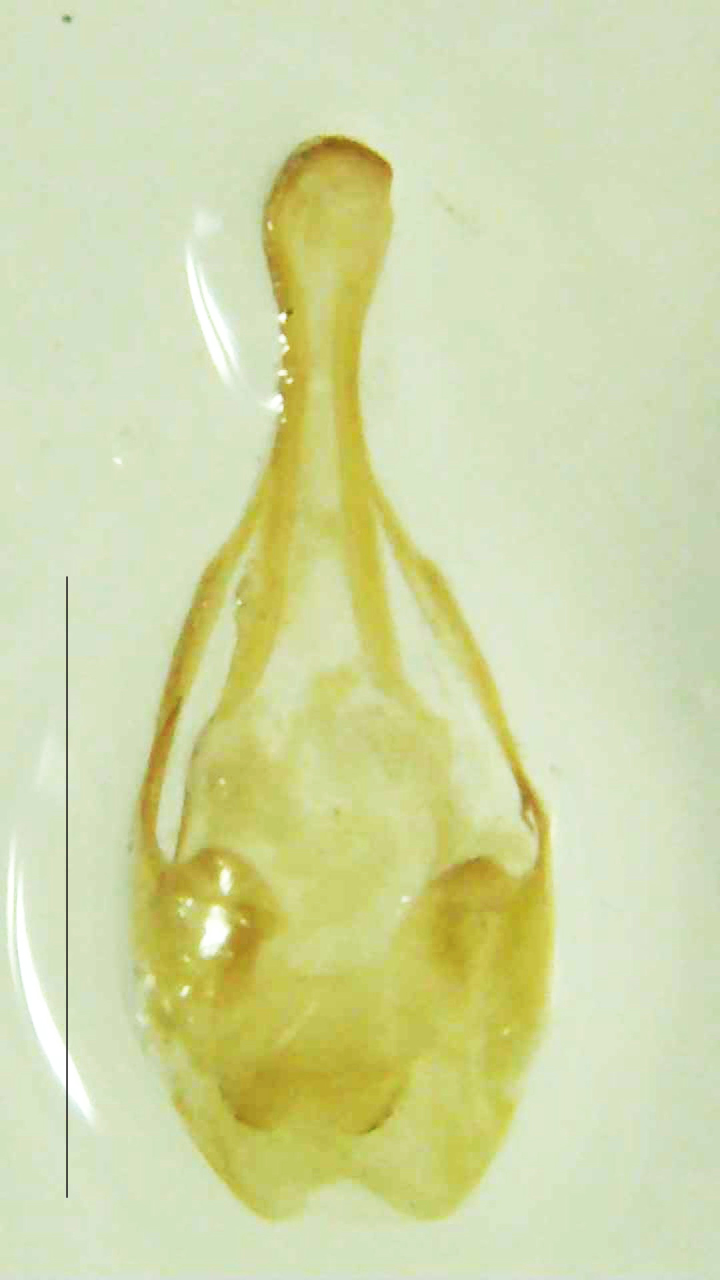
Aedeagal sheath ventral. Scale bar: 0.5 mm;

**Figure 5c.**
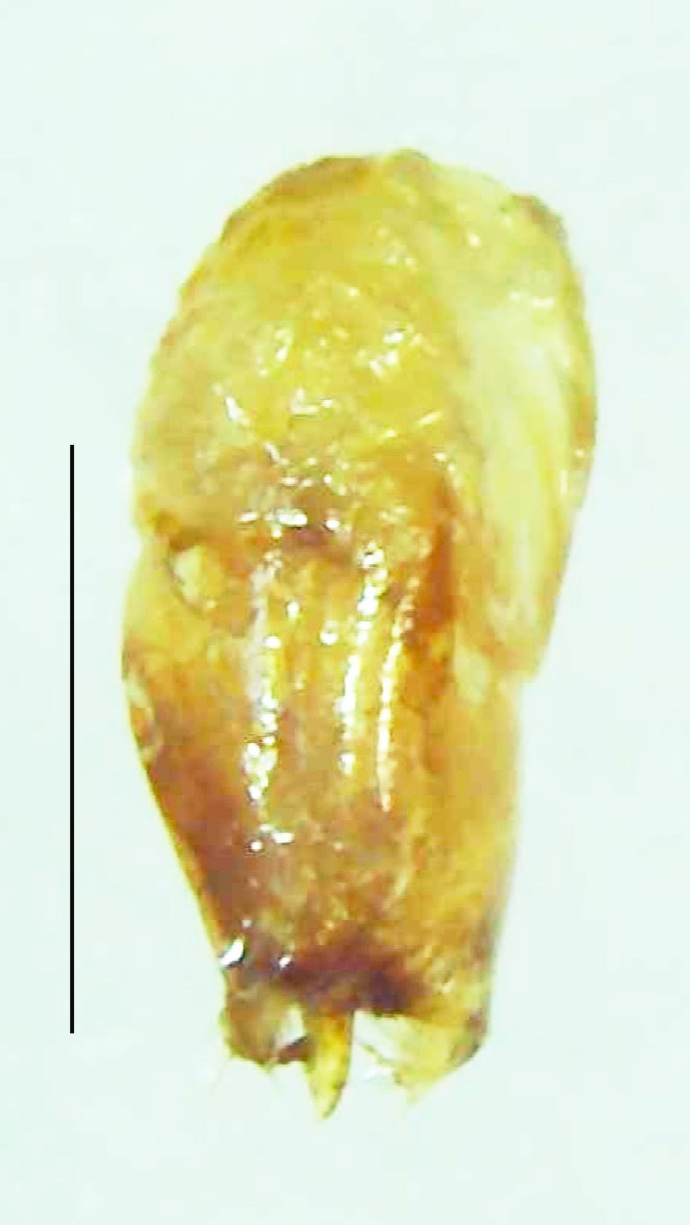
Aedeagus dorsal. Scale bar: 0.5 mm;

**Figure 5d.**
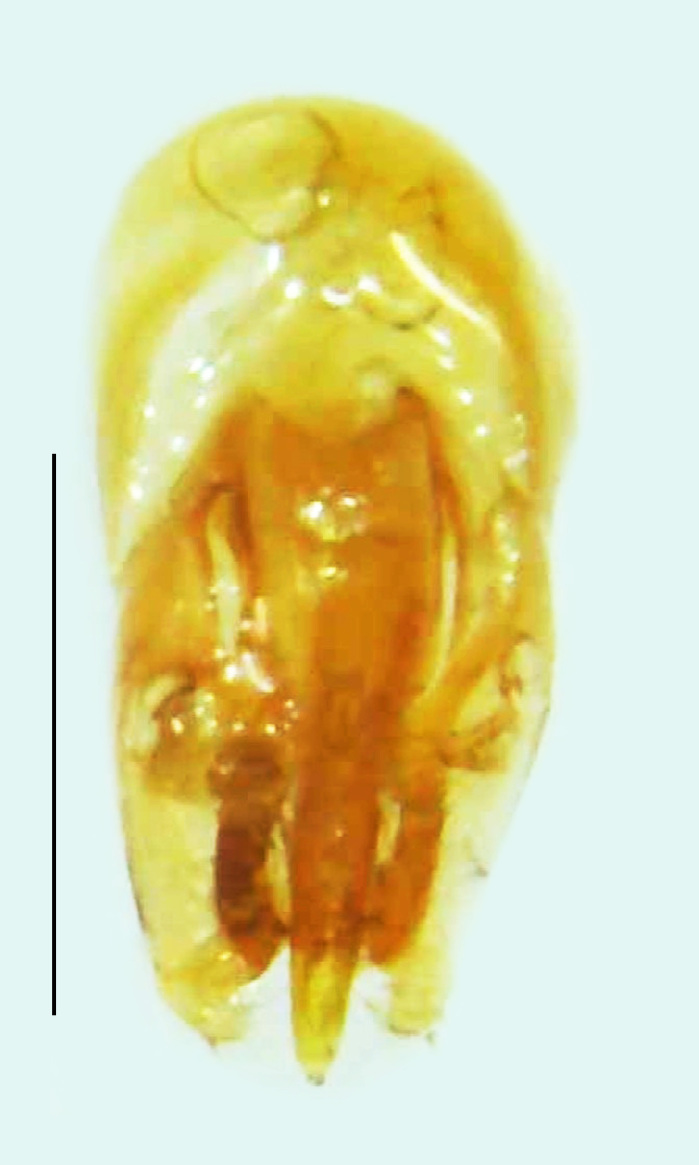
Aedeagus ventral. Scale bar: 0.5 mm.

**Figure 6a.**
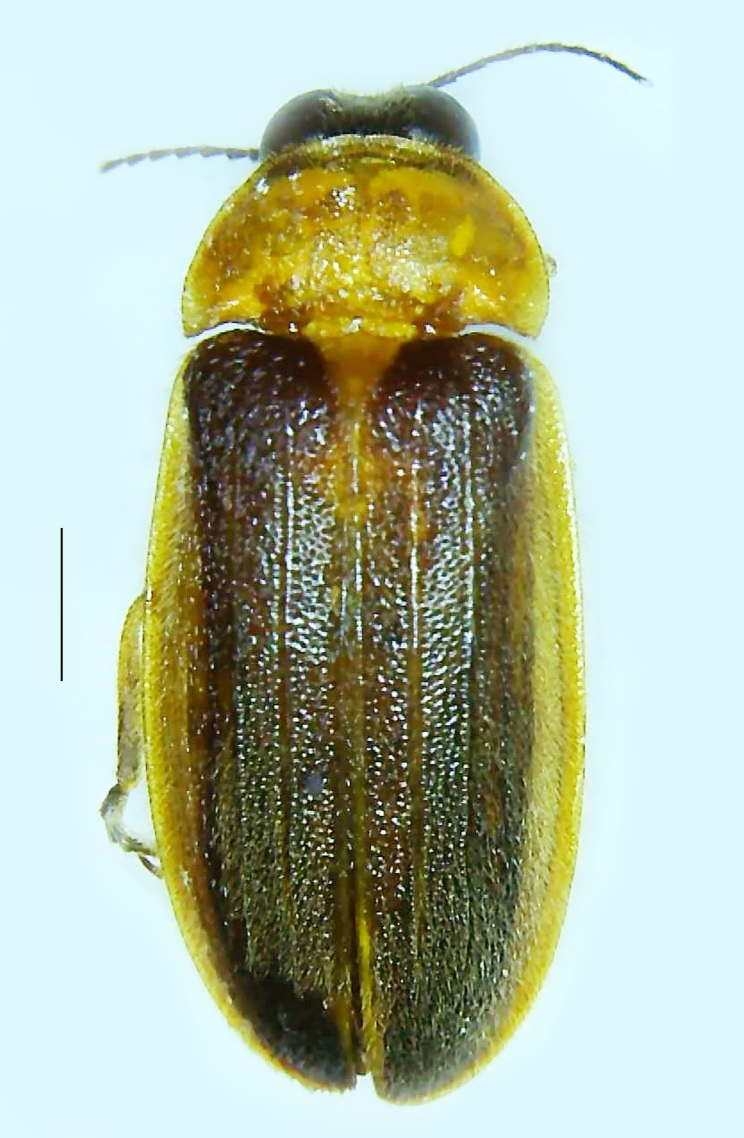
Male dorsal habitus. Scale bar: 1 mm;

**Figure 6b.**
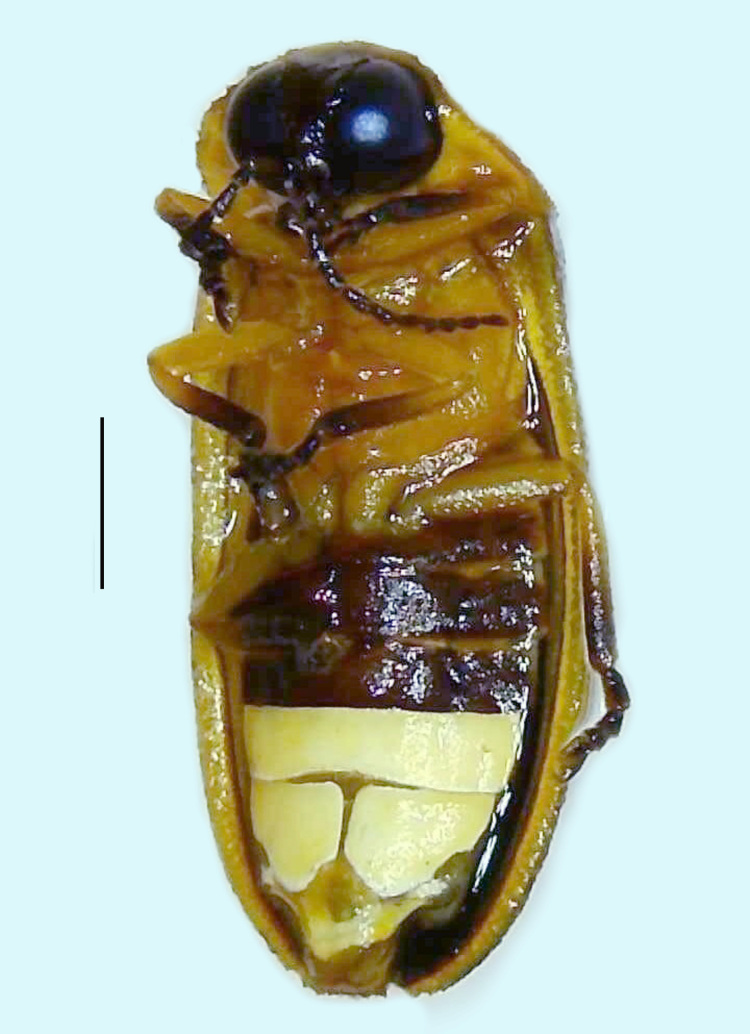
Male ventral habitus. Scale bar: 1 mm.

**Figure 7a.**
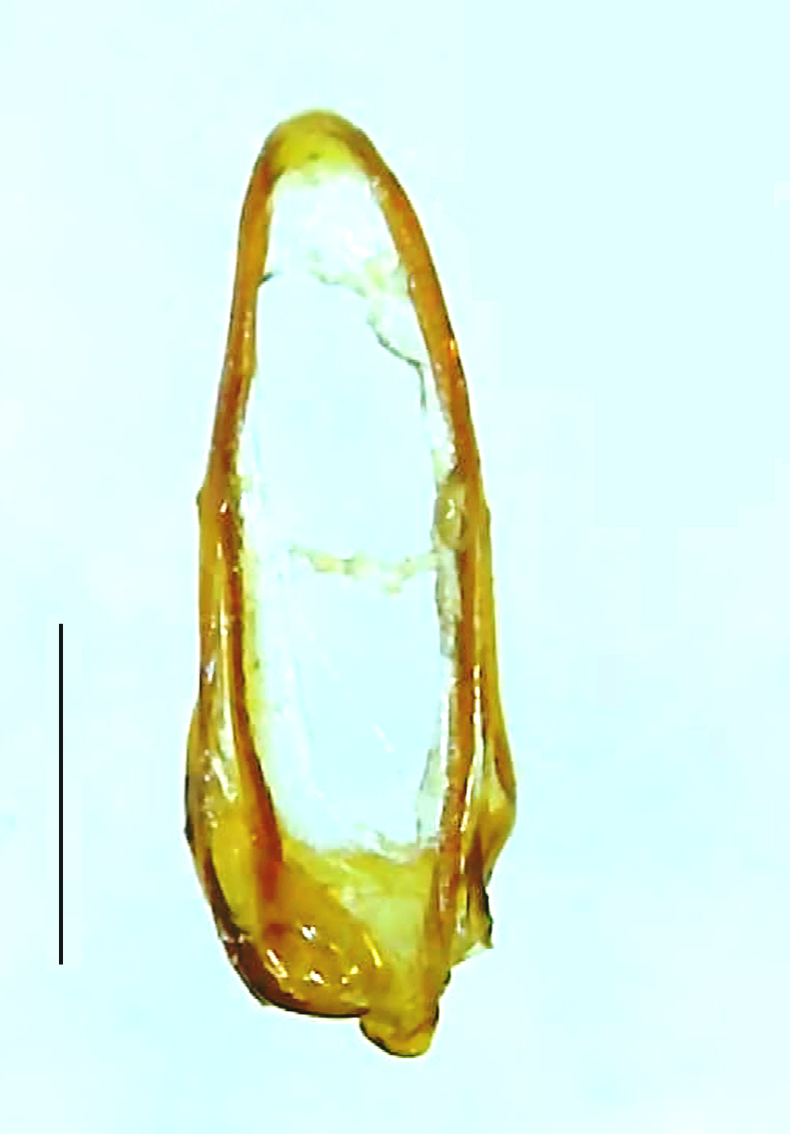
Aedeagal sheath dorsal. Scale bar: 0.5 mm;

**Figure 7b.**
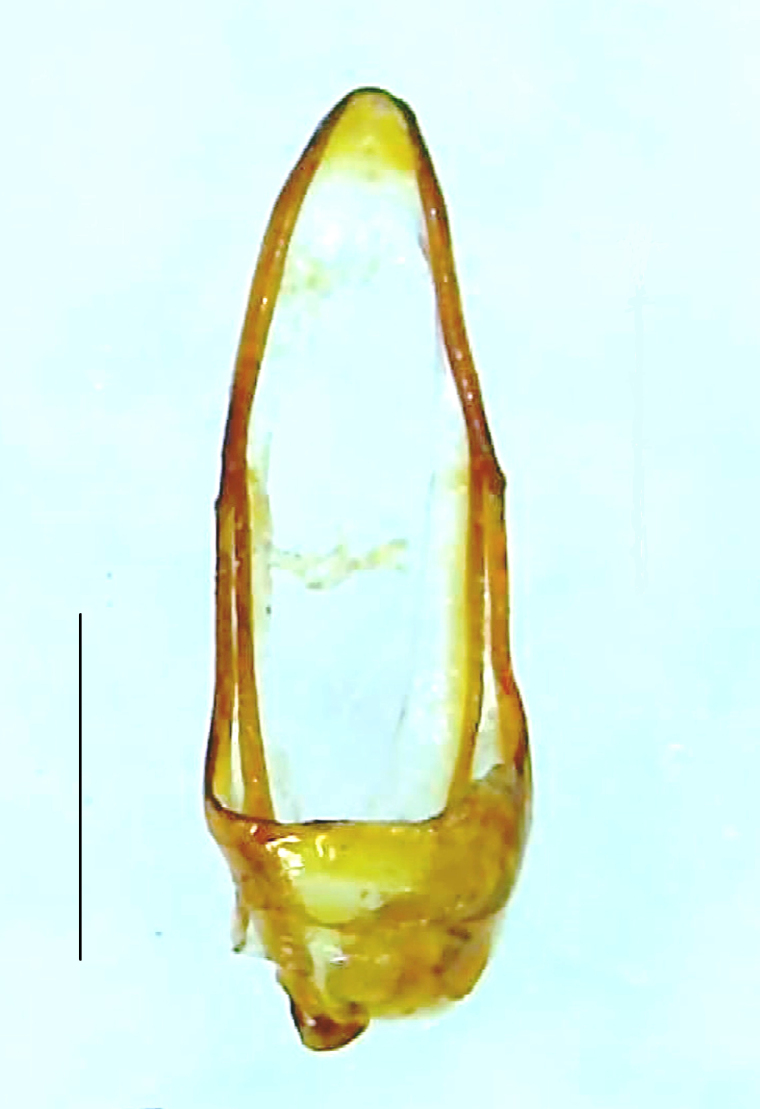
Aedeagal sheath ventral. Scale bar: 0.5 mm;

**Figure 7c.**
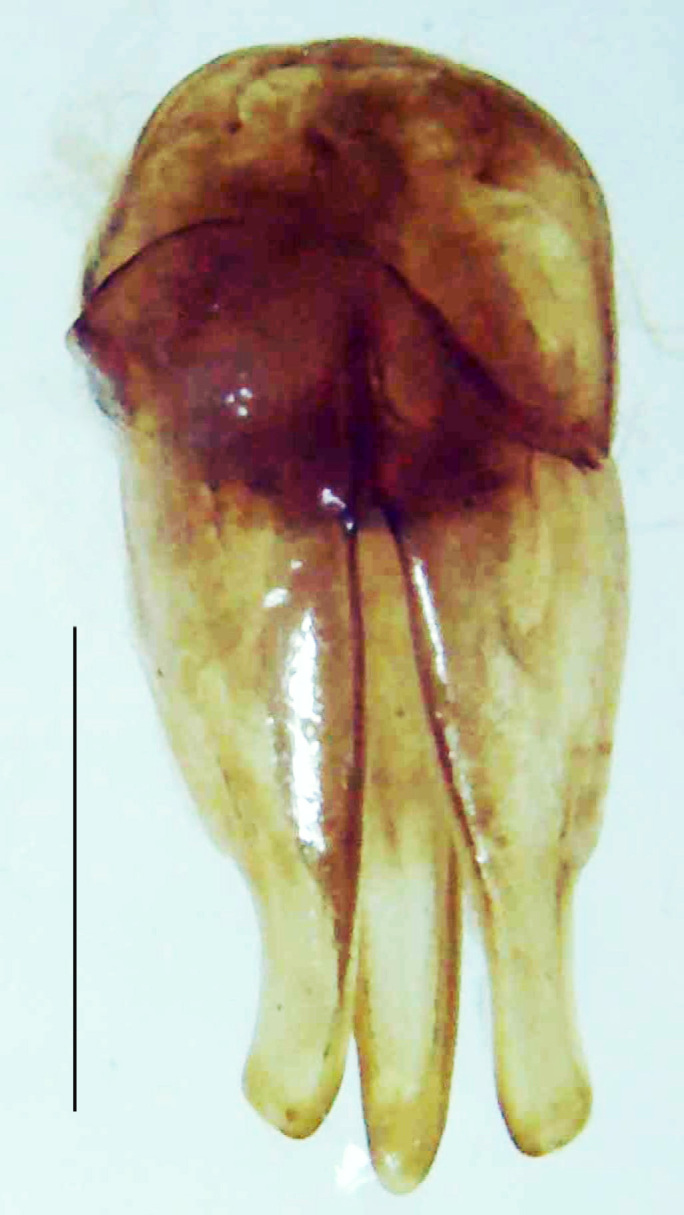
Aedeagus dorsal. Scale bar: 0.5 mm;

**Figure 7d.**
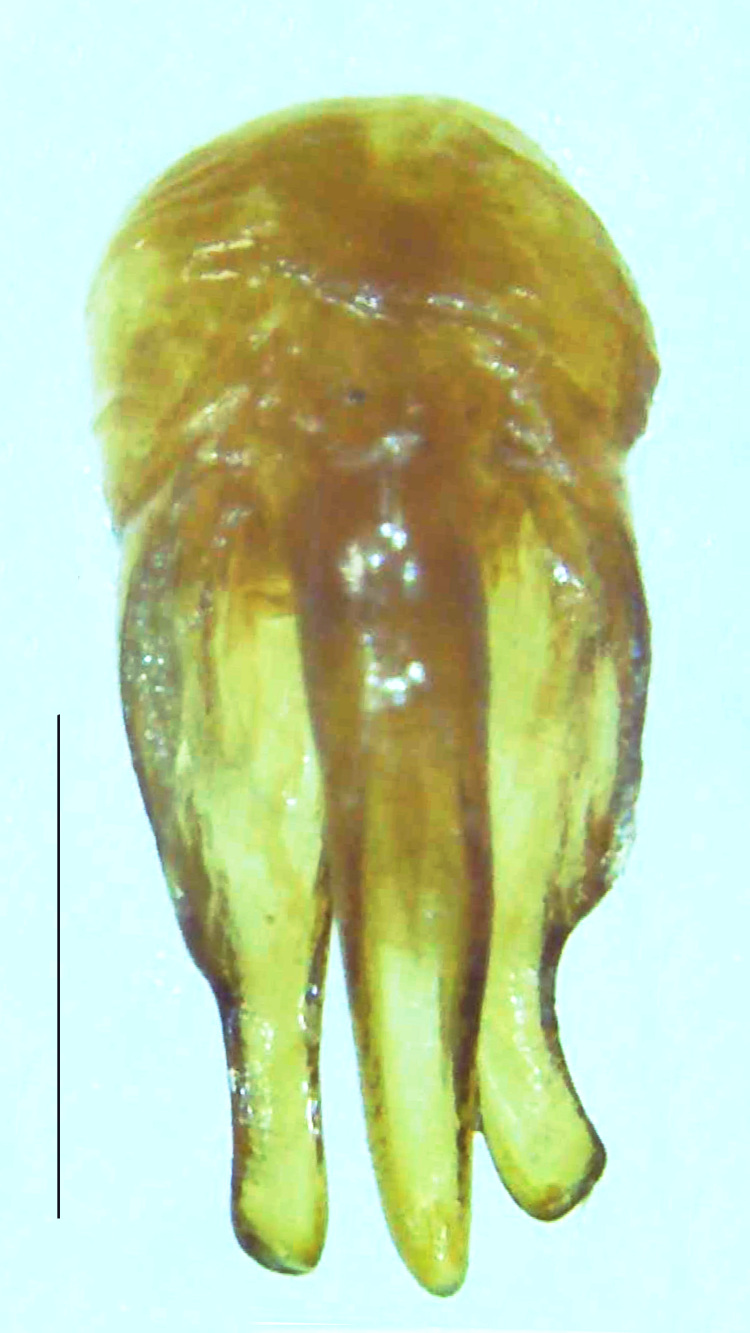
Aedeagus ventral. Scale bar: 0.5 mm.

**Figure 8a.**
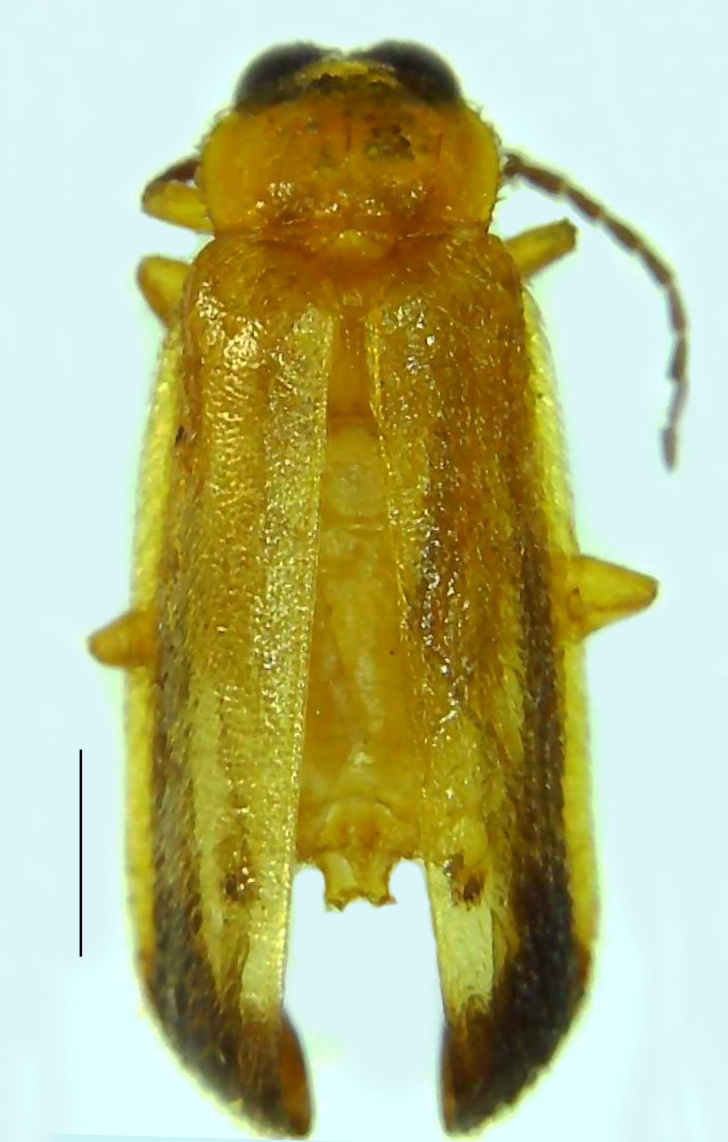
Male dorsal habitus. Scale bar: 1 mm;

**Figure 8b.**
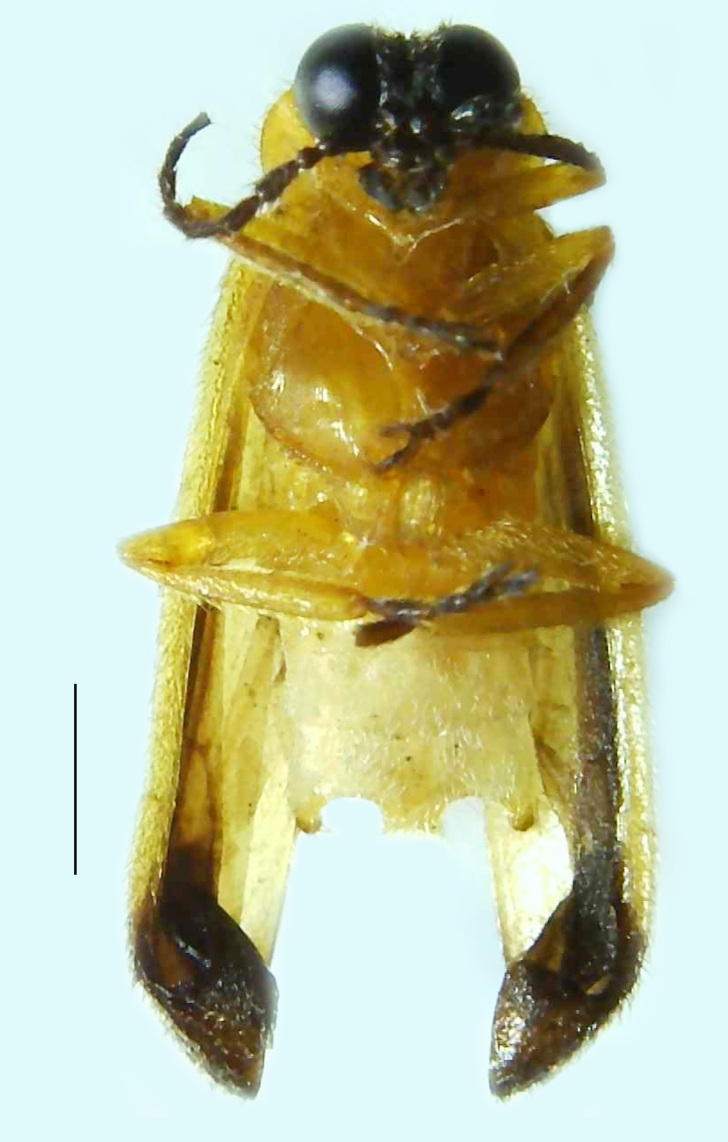
Male ventral habitus. Scale bar: 1 mm.

**Figure 9a.**
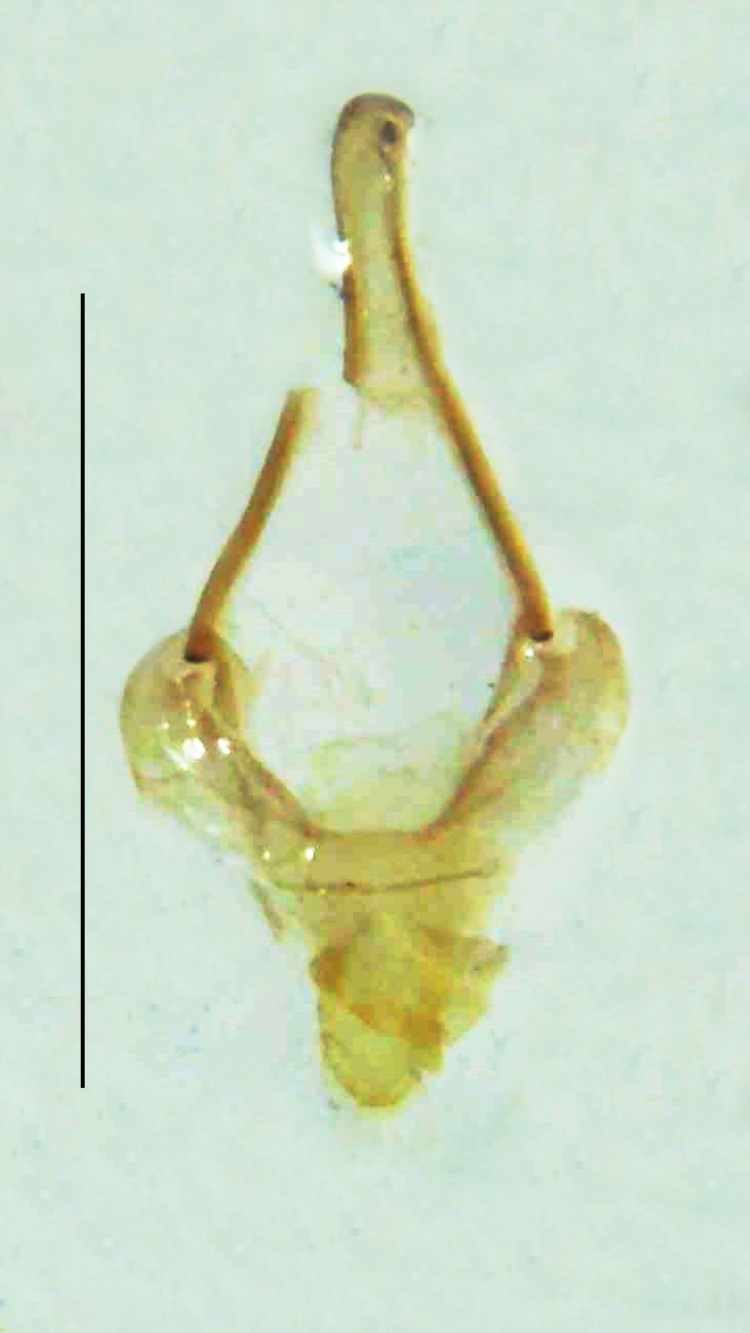
Aedeagal sheath dorsal. Scale bar: 0.5 mm;

**Figure 9b.**
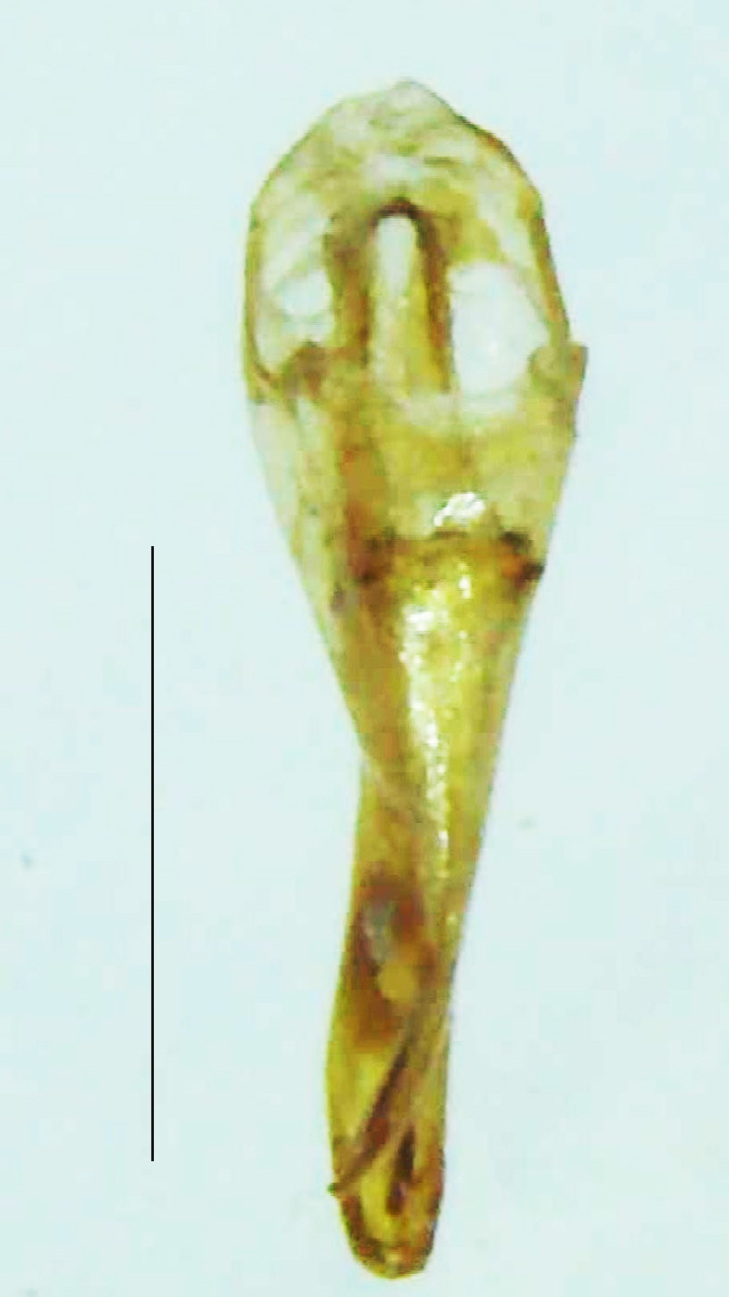
Aedeagus dorsal. Scale bar: 0.5 mm;

**Figure 9c.**
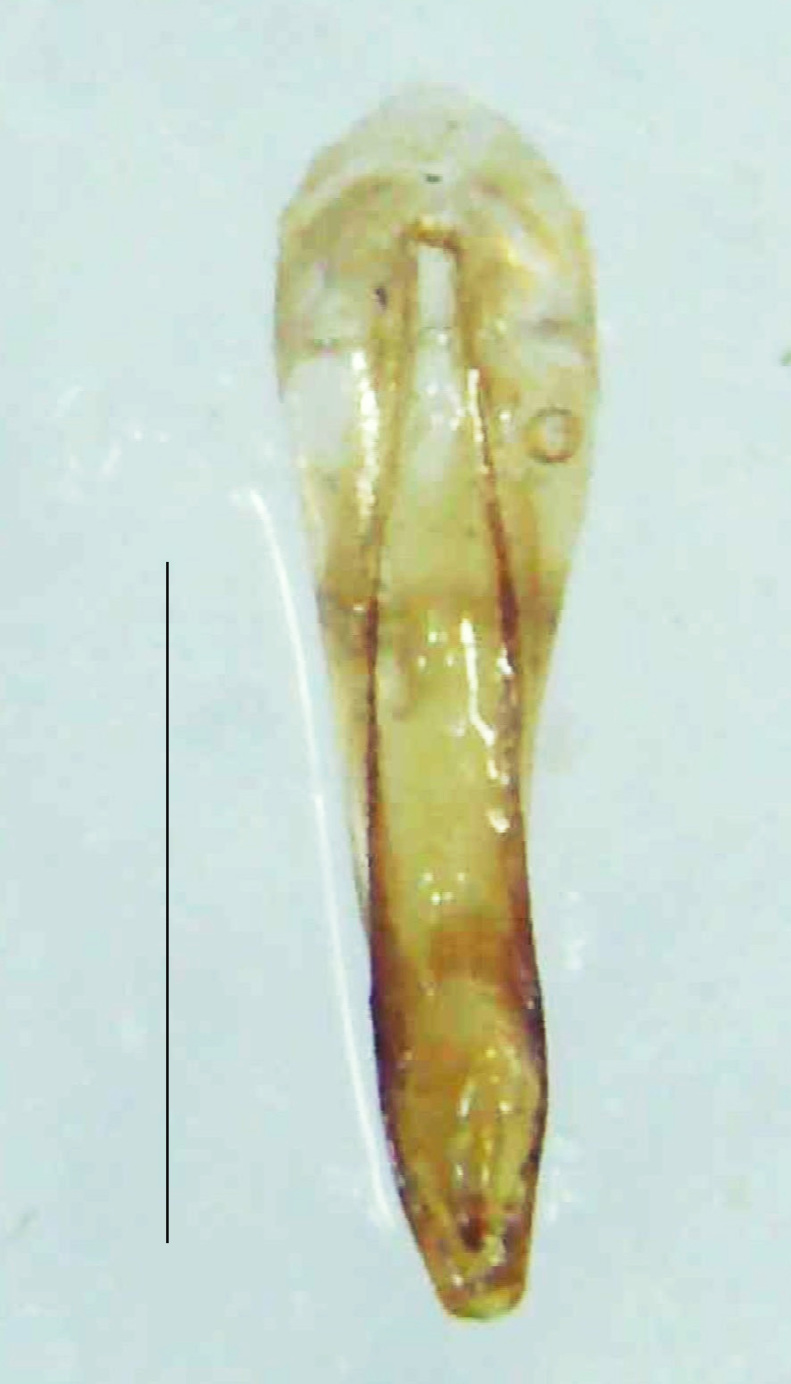
Aedeagus ventral. Scale bar: 0.5 mm;

**Figure 9d.**
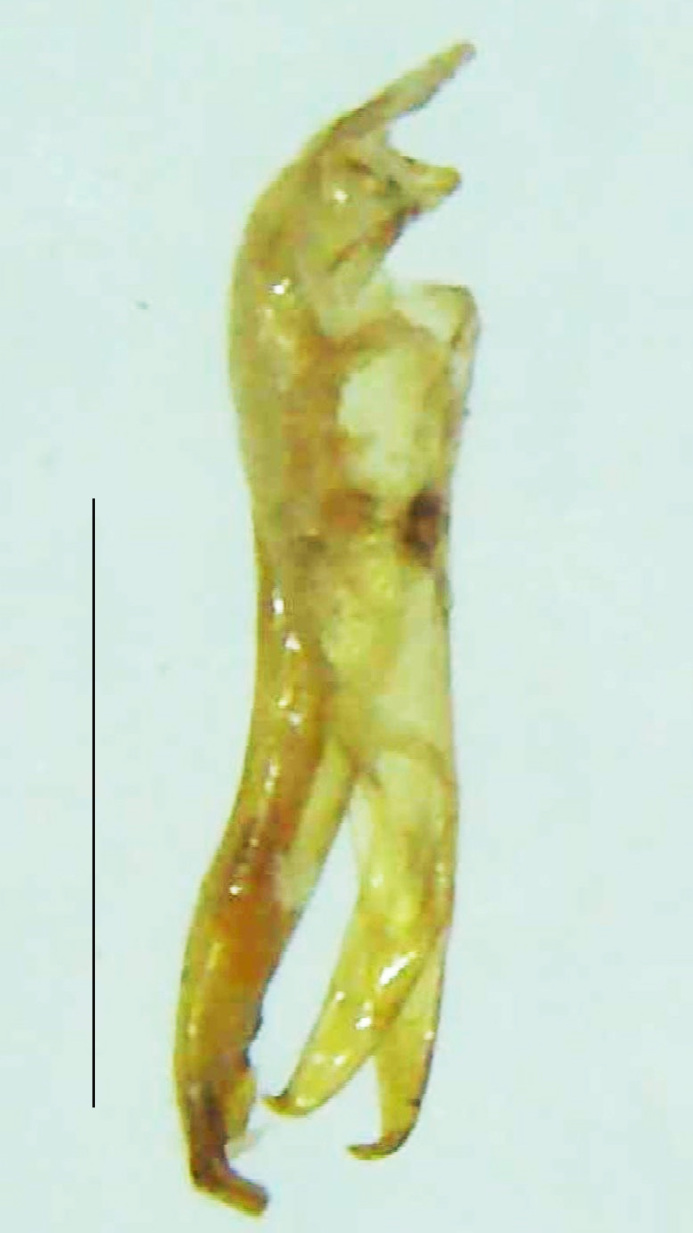
Aedeagus lateral. Scale bar: 0.5 mm.

**Figure 10a.**
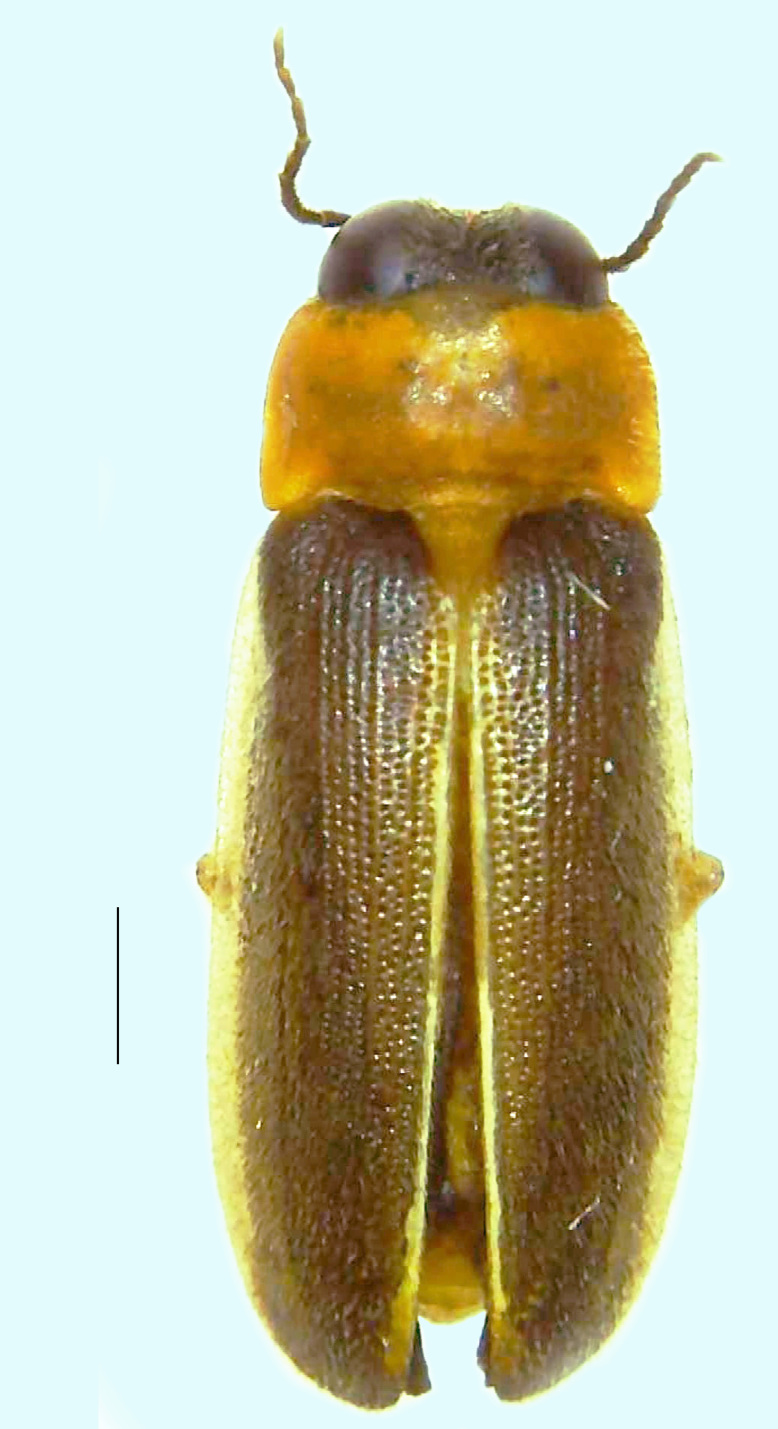
Male dorsal habitus. Scale bar: 1 mm;

**Figure 10b.**
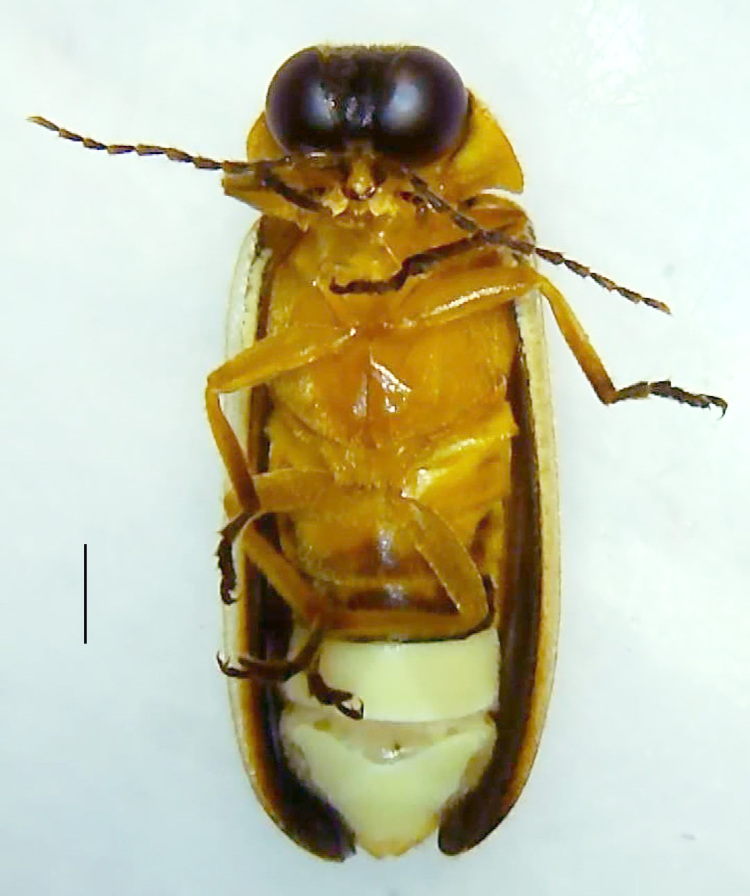
Male ventral habitus. Scale bar: 1 mm;

**Figure 10c.**
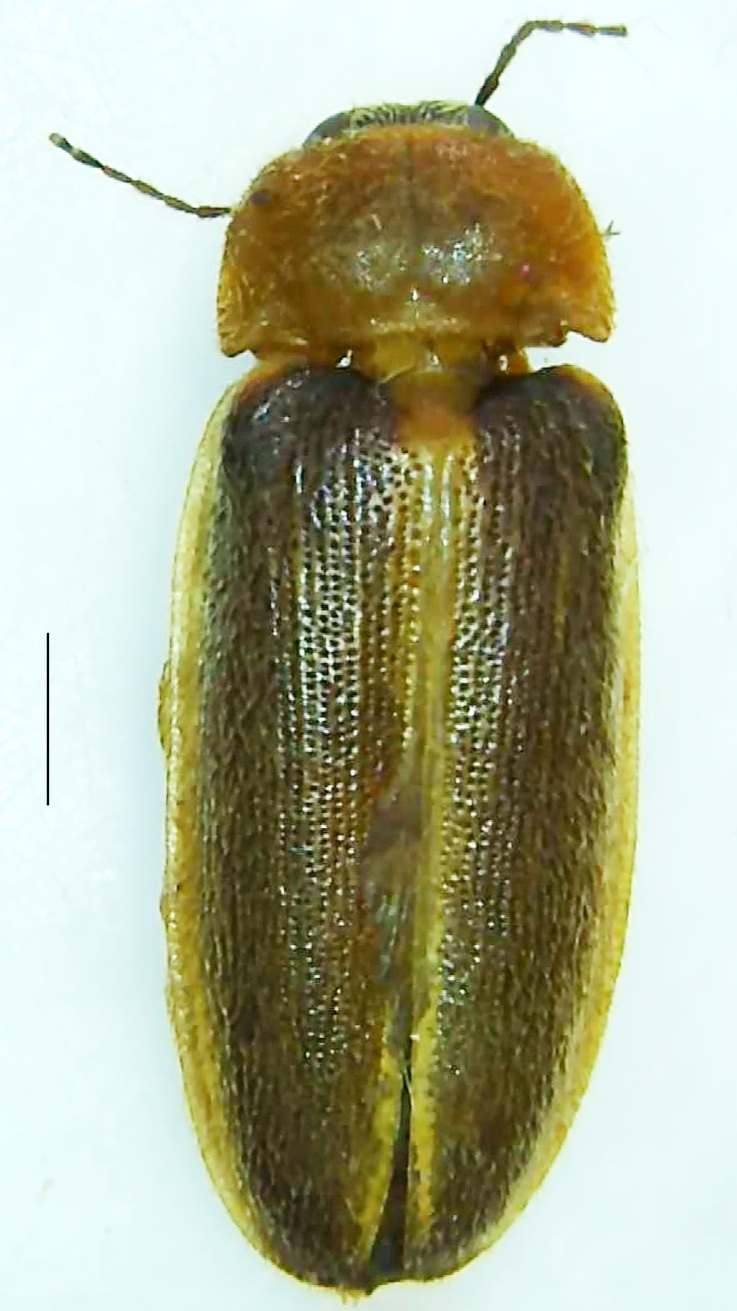
Female dorsal habitus. Scale bar: 1 mm;

**Figure 10d.**
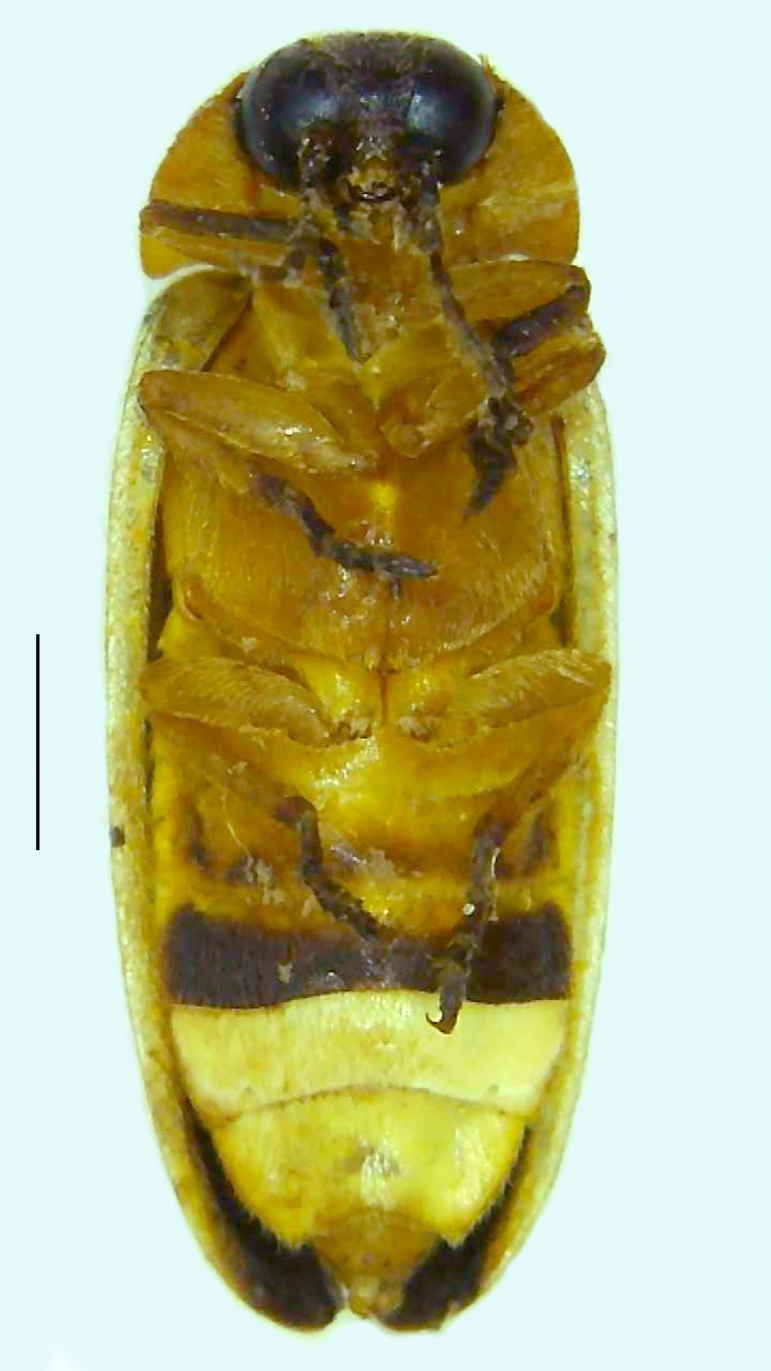
Female ventral habitus. Scale bar: 1 mm.

**Figure 11a.**
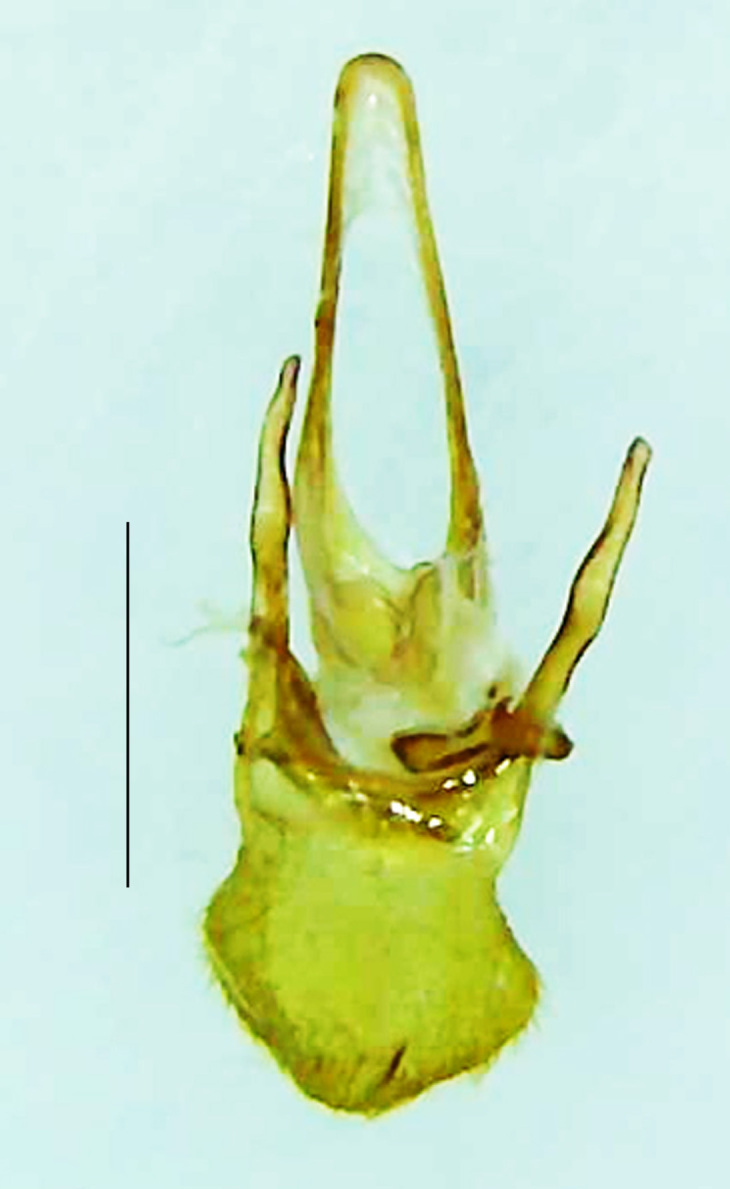
Aedeagal sheath dorsal, tergite 8 dorsal and two of the three sclerites that surround the sheath in a band of muscle. Scale bar 0.5 mm;

**Figure 11b.**
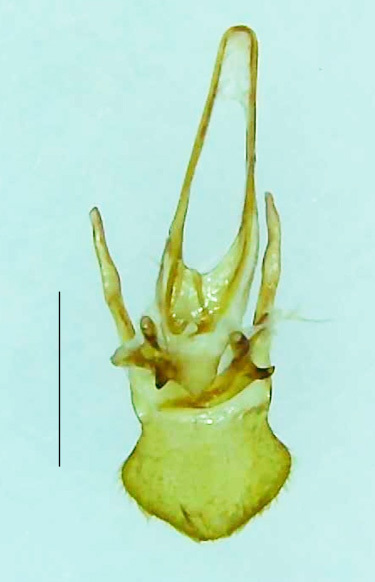
Aedeagal sheath ventral, tergite 8 ventral and two of the three sclerites that surround the sheath in a band of muscle. Scale bar 0.5 mm;

**Figure 11c.**
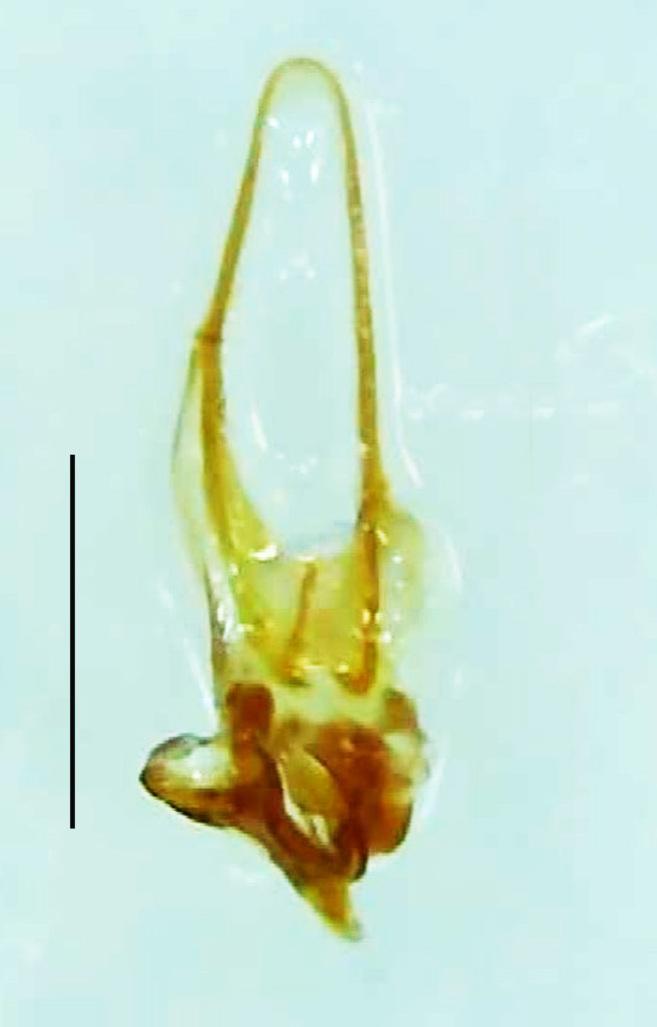
Aedeagal sheath ventral and two of the sclerites. Scale bar 0.5 mm;

**Figure 11d.**
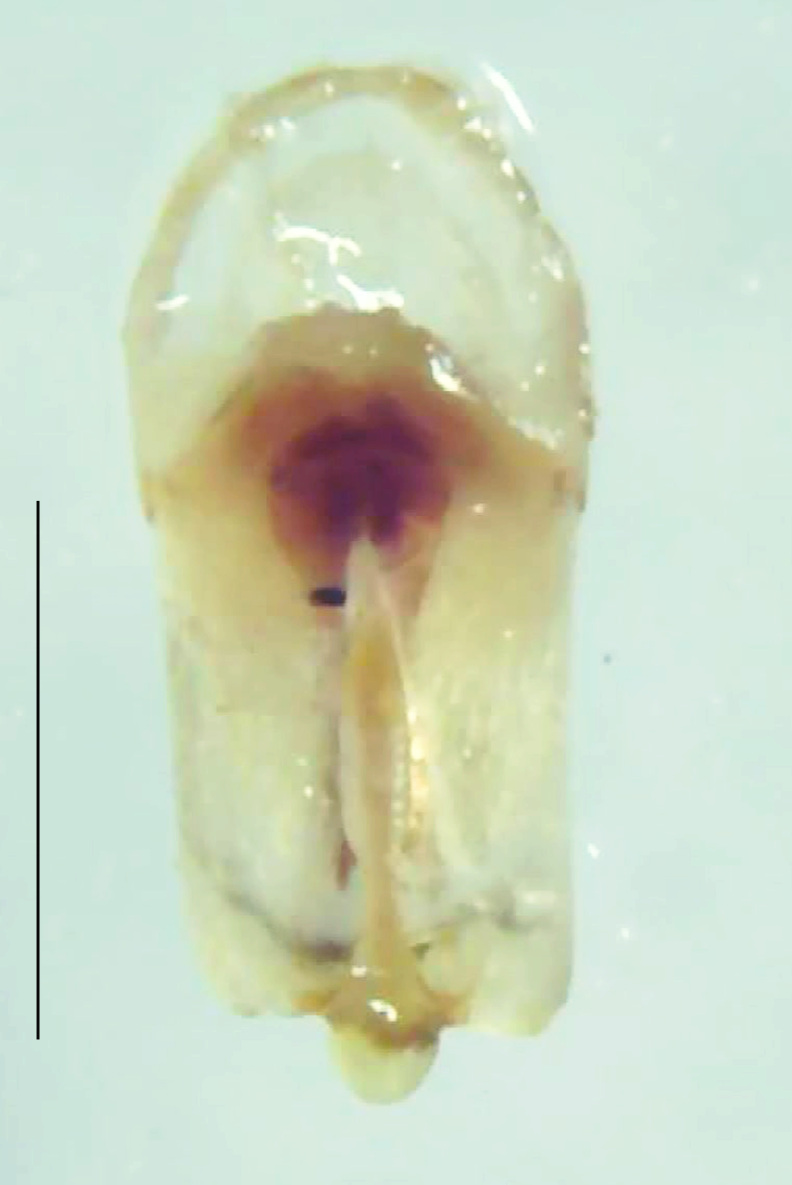
Aedeagus dorsal. Scale bar 0.5 mm;

**Figure 11e.**
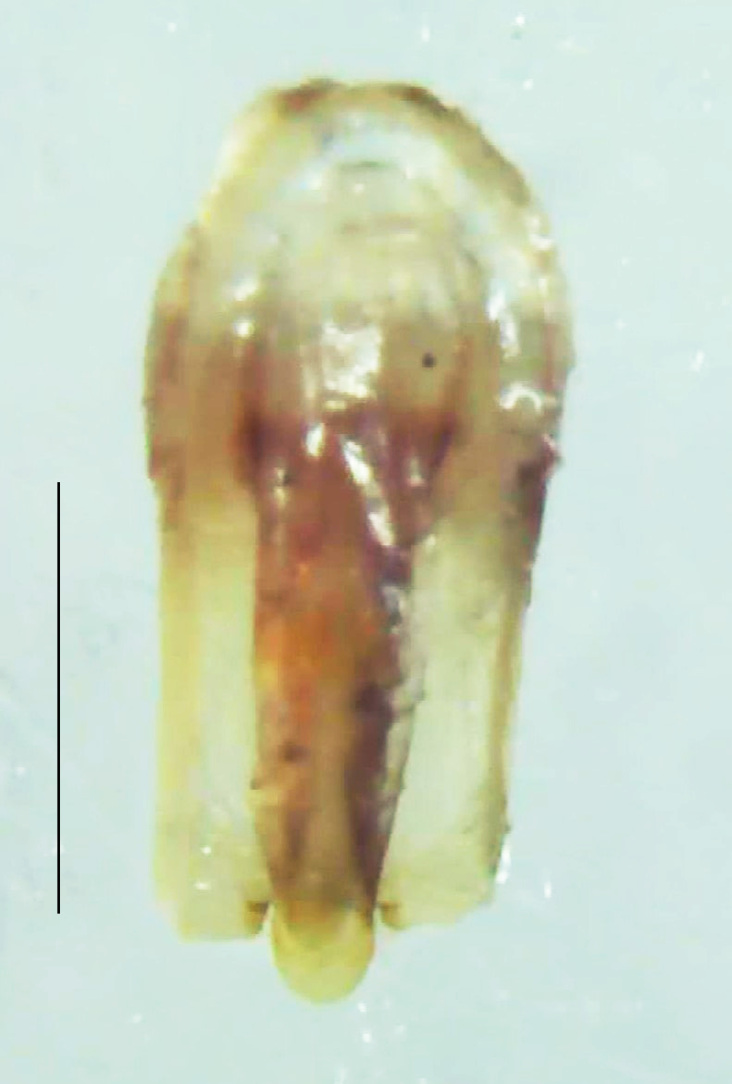
Aedeagus ventral. Scale bar 0.5 mm.

**Figure 12a.**
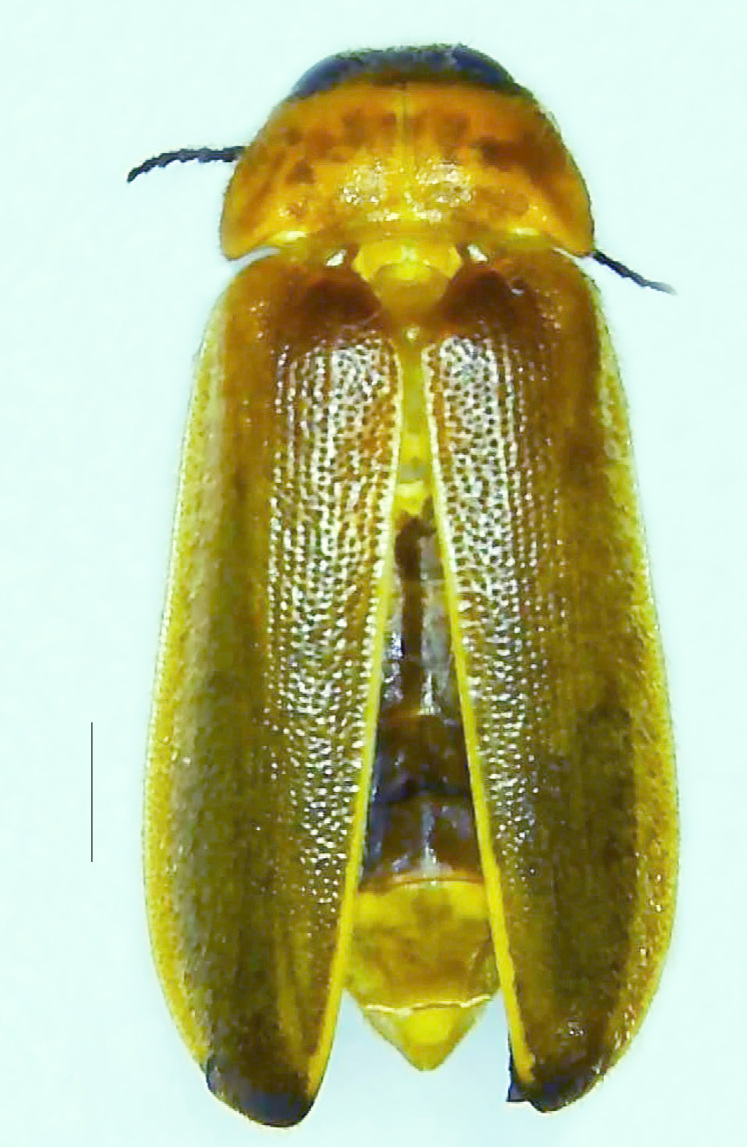
Male dorsal habitus. Scale bar: 1 mm;

**Figure 12b.**
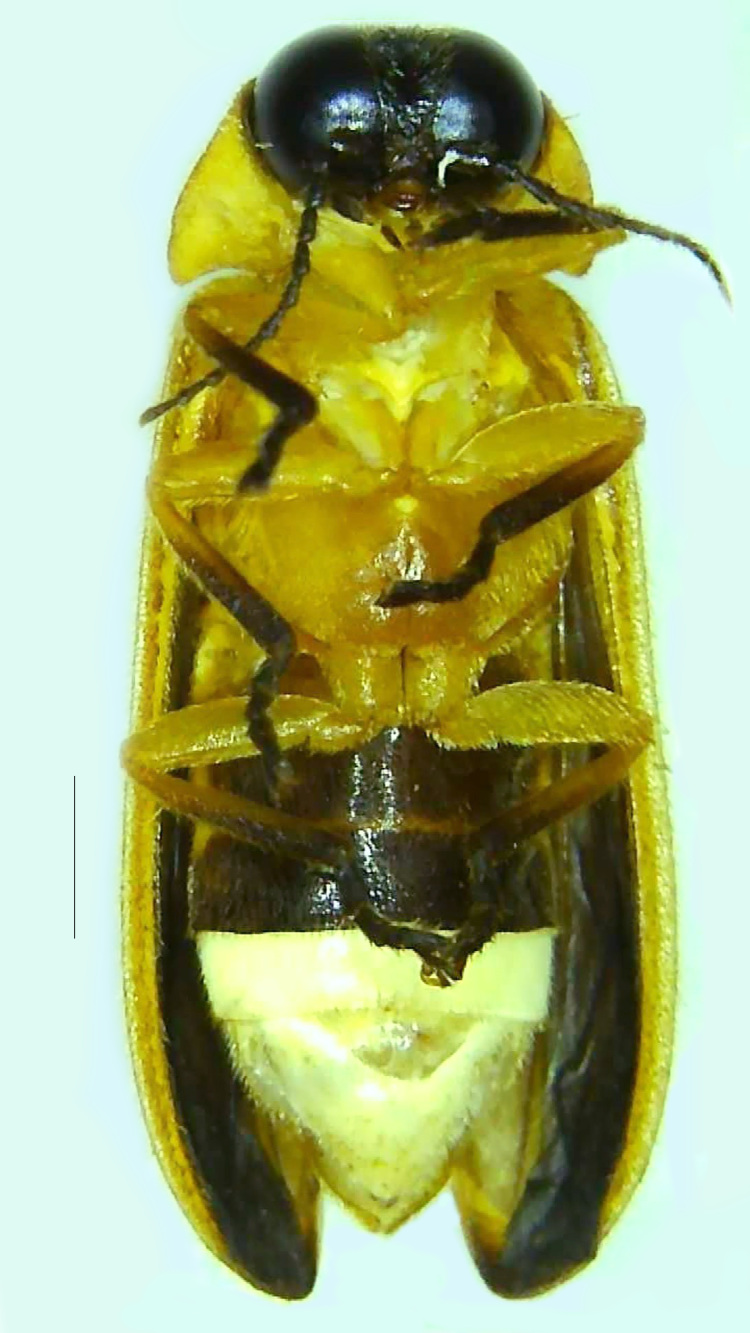
Male ventral habitus. Scale bar: 1 mm.

**Figure 13a.**
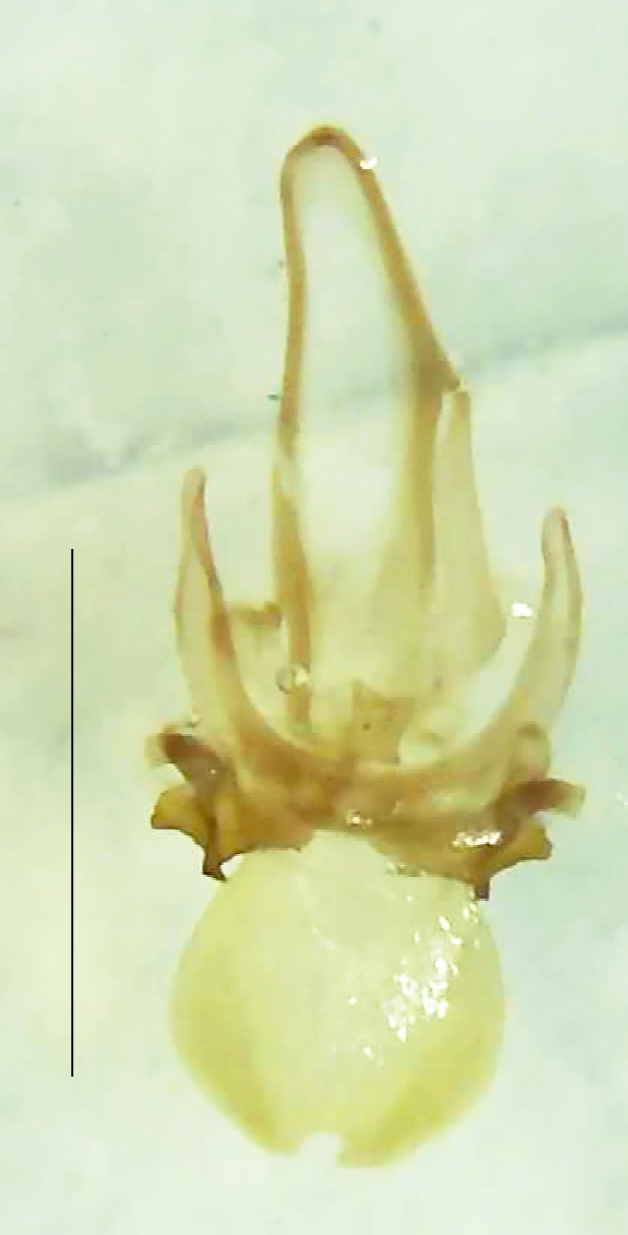
Aedeagal sheath dorsal (partly broken near the junction of sheath tergite and sternite), tergite 8 dorsal and two of the three sclerites. Scale bar: 0.5 mm;

**Figure 13b.**
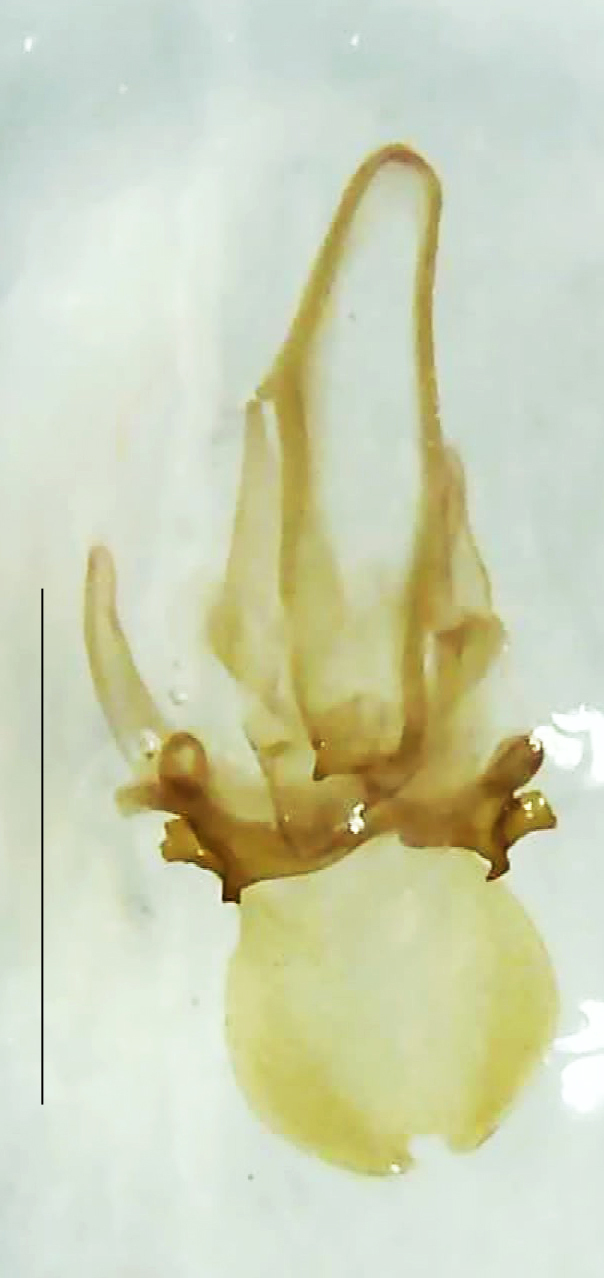
Aedeagal sheath ventral (partly broken near the junction of sheath tergite and sternite), tergite 8 ventral and two of the sclerites. Scale bar: 0.5 mm;

**Figure 13c.**
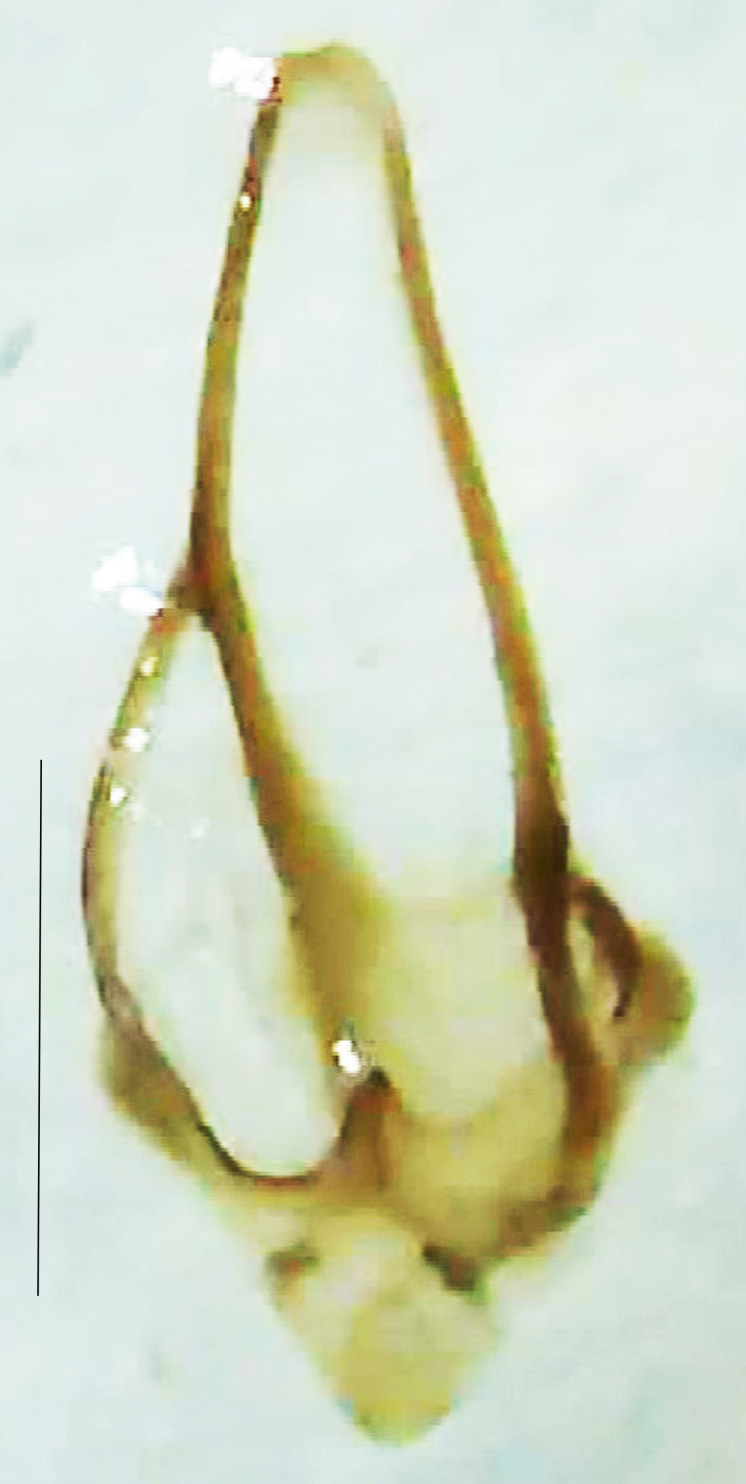
Aedeagal sheath dorsal. Scale bar: 0.5 mm;

**Figure 13d.**
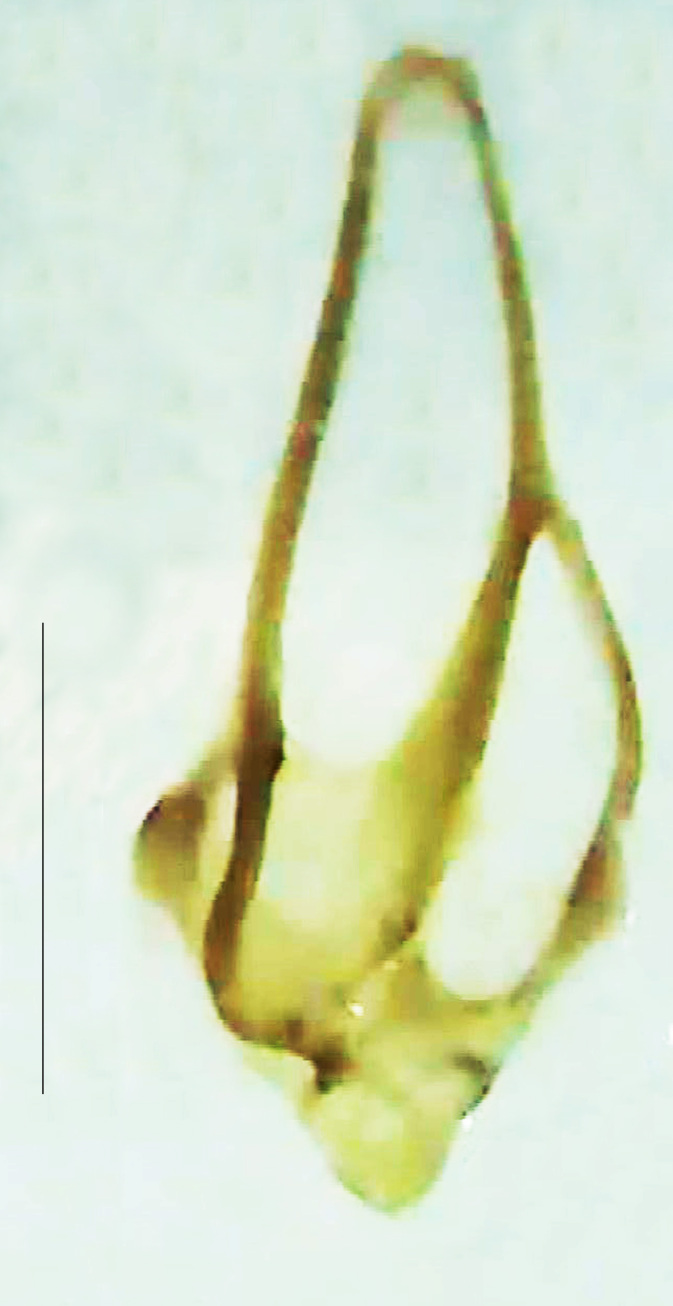
Aedeagal sheath ventral. Scale bar: 0.5 mm;

**Figure 13e.**
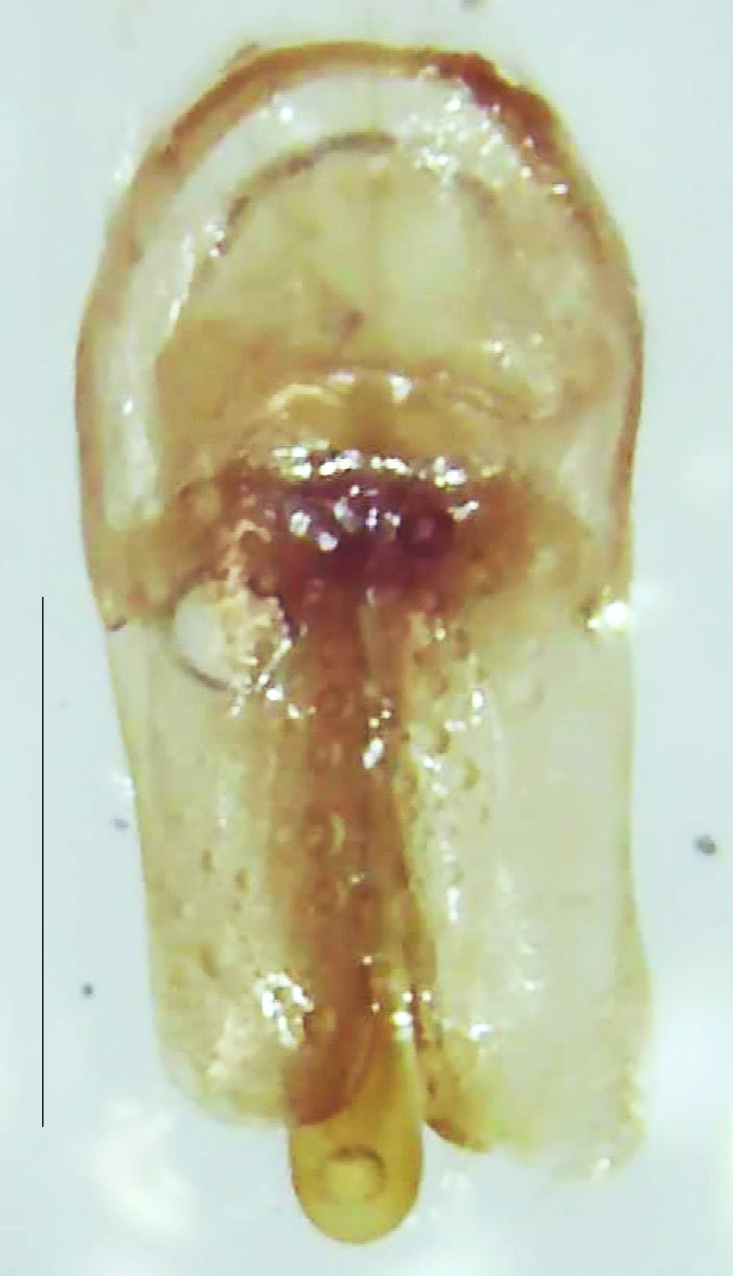
Aedeagus dorsal. Scale bar: 0.5 mm;

**Figure 13f.**
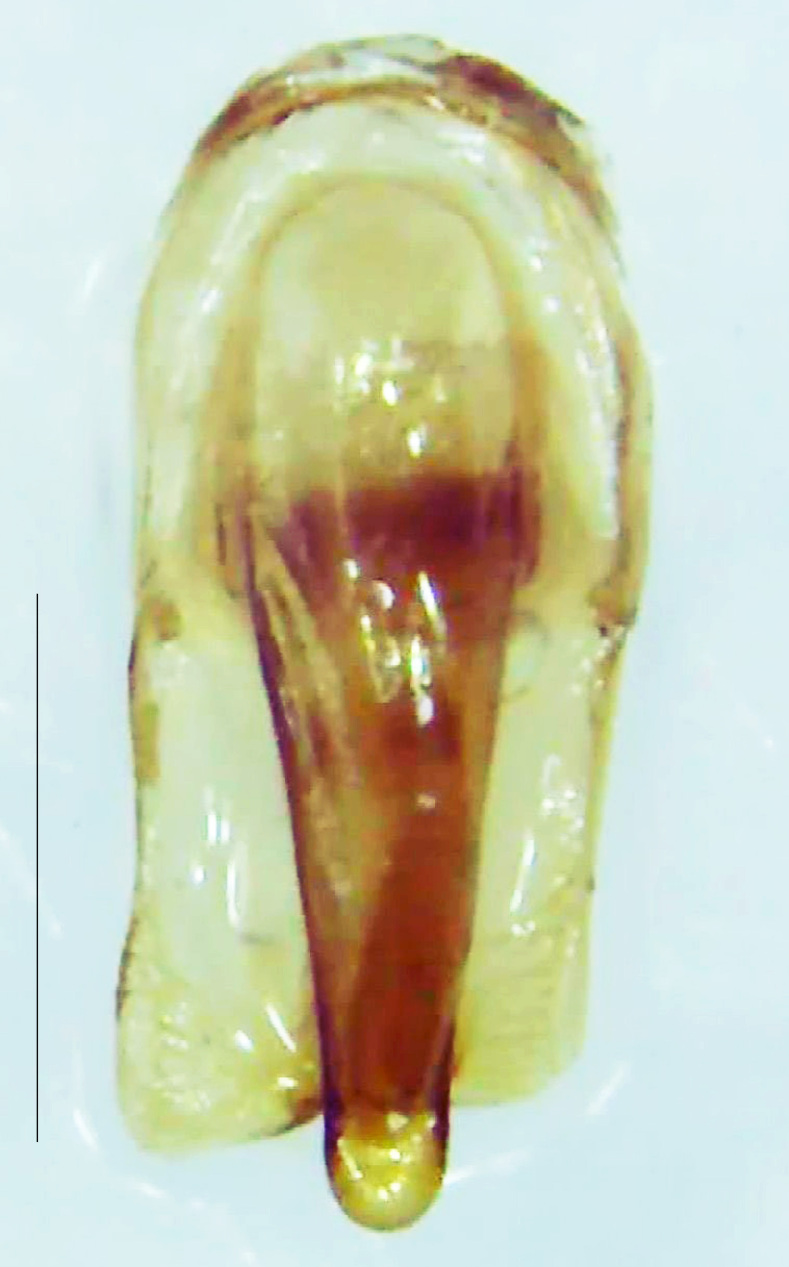
Aedeagus ventral. Scale bar: 0.5 mm.

**Table 1.** **Checklist of Indian Lucioline firefly species (for synonymy, see** Ballantyne et al. (2019)) . [^##^ Listed as Species Incertae Sedis in Ballantyne et al. (2019), ** Defined as *Luciola* s. lato in Ballantyne et al. (2019), ^#^ Recommended for transfer to *Curtos* in Ballantyne et al. (2019), * Defined as Luciola s. str. Laporte in Ballantyne et al. (2019) , *^£^* Recommended for transfer to *Curtos* in Ballantyne and Jusoh (2016), India (?) - indicates precise location in India not mentioned.

**Sl. No.**	**Genus and species**	**Records from India**	**Records from elsewhere**
1	*Absconditachinensis* (L., 1767)	Assam; Bihar; Maharashtra (Chen 2003, Ballantyne et al. 2013, Ballantyne et al. 2019, Fu 2014). Tamilnadu - Madras; Maharashtra - Bombay; Bengal (Gorham 1880); Karnataka - Belgaum, Kanara, Madura (Gorham 1895); Odisha - herein.	China - mainland; Thailand (Chen 2003, Fu 2014, Ballantyne et al. 2019). Cambodia; Myanmar - Rangoon, Tharawaddy (Gorham 1895). Sri Lanka; Thailand; Vietnam (McDermott 1966, Ballantyne et al. 2016, Wattanachaiyingcharoen et al. 2016, Wijekoon et al. 2016, Seri and Rahman 2021).
2	*Absconditaperplexa* (Walker, 1858)	Assam; Madhya Pradesh (Ballantyne et al. 2013). Assam - Guwahati (Ballantyne et al. 2019); Odisha - herein, West Bengal - herein.	Sri Lanka; Thailand (Ballantyne et al. 2019). Cambodia (Ballantyne et al. 2016, Seri and Rahman 2021). Sri Lanka (Wijekoon et al. 2021).
3	*Absconditaterminalis* (Olivier, 1883)	West Bengal; Odisha (Ghosh et al. 2021).	Cambodia; China - mainland; Laos; Thailand; Vietnam (Chen 2003, Yiu 2012, Ballantyne et al. 2013, Ballantyne et al. 2019, Fu 2014). Thailand; Vietnam (Olivier 1905, McDermott 1966).
4	*Asymmetricatacircumdata* (Motschulsky, 1854)	Indes orientales (Motschulsky 1854). India (?) (McDermott 1966). Meghalaya - Garo hills (Rabha and Gohain Barua 2016).	Cambodia; Laos; Myanmar; Singapore; Thailand (Lloyd et al. 1989,Nak-eiam et al. 2011). Vietnam (Ballantyne and Lambkin 2009, Fu et al. 2012b, Ballantyne et al. 2019). Thailand - Bangkok (Theraphat 1969).
5	*Asymmetricatahumeralis* (Walker, 1858)	Goa - Mormugao; Maharashtra - Malabar Hills; Karnataka; Tamilnadu; Kerala - Nilgiri hills (Ballantyne et al. 2019).	Indonesia - Java, Sumatra; Sri Lanka (Ballantyne et al. 2019). Sri Lanka (Olivier 1905, McDermott 1966, Wijekoon et al. 2016, Wijekoon et al. 2021).
6	*Asymmetricataovalis* (Hope, 1831)	Goa; West Bengal - Kalimpong (Ballantyne and Lambkin 2009, Ballantyne et al. 2019). Assam (Gorham 1880). North West India; Karnataka - Belgaum, Kanara (Gorham 1895). Odisha - herein.	Myanmar; Nepal; Sri Lanka; Thailand (Ballantyne et al. 2019). Indonesia - Sumatra; Myanmar - Burma, Rangoon, Tharawaddy; Nepal; Vietnam - Tonkin (Gorham 1895, McDermott 1966). Thailand (Wattanachaiyingcharoen et al. 2016, Pronak et al. 2018, Seri and Rahman 2021).
7	*Curtosacerra* (Gorham, 1895)	India (Ballantyne et al. 2019). Karnataka - Belgaum, Kanara (Gorham 1895). India (?) (McDermott 1966, Jeng et al. 1998, Fu et al. 2012a).	
8	*Curtosvariolosus* (Bourgeois, 1907)	India (?) (Ballantyne et al. 2019, McDermott 1966, Jeng et al. 1998, Fu et al. 2012a).	
9	*Inflataindica* (Motschulsky, 1854)	Andaman Island; Maharashtra - Mumbai (Gorham 1880, McDermott 1966). Indes Orientales (Olivier 1905). Maharashtra - Mumbai (Raj 1947).	
10	*Luciolaaurantiaca* Pic, 1927 ^##^	Southern India (McDermott 1966). India (?) (Ballantyne et al. 2019).	
11	*Luciolaauritula* Olivier, 1910 **	India (?) (Ballantyne et al. 2019).	Sri Lanka (McDermott 1966).
12	*Luciolacomplanata* Gorham, 1895 ^#^	Karnataka - Kanara (Gorham 1895). India (?) (Ballantyne et al. 2019).	Madagascar (McDermott 1966).
13	*Luciolagigas* Olivier, 1888 **	West Bengal - Kolkata (Ballantyne et al. 2019). India (?) (Olivier 1905, McDermott 1966).	Madagascar (McDermott 1966).
14	*Luciolahorni* Bourgeois, 1905 *	Tamil Nadu - Tanjore (McDermott 1966, Ballantyne et al. 2019)	Sri Lanka (Olivier 1905, McDermott 1966, Ballantyne et al. 2019, Wijekoon et al. 2012, Wijekoon et al. 2021).
15	*Luciolamaindroni* Pic, 1927	Maharashtra - Malabar (McDermott 1966).	
16	*Luciolamulticostulata* Pic, 1927 ^#^	India - southern part (McDermott 1966). India (?) (Ballantyne et al. 2019).	
17	*Luciolanigripes* Gorham, 1903 ^#^	Tamilnadu (Gorham 1903, Olivier 1905, McDermott 1966). India (?) (Ballantyne et al. 2019).	
18	*Luciolanotaticollis* Pic 1914 ^£^	Tamil Nadu - Madura (Ballantyne et al. 2016, Ballantyne et al. 2019).	Indonesia (Ballantyne et al. 2016, Ballantyne et al. 2019). East Indies - Indonesian Archipelago, Malaysian Borneo, the Philippine Archipelago and New Guinea (McDermott 1966).
19	*Luciolaobscura* Pic, 1928 **	India (?) (Ballantyne et al. 2016, Ballantyne et al. 2019).	Indonesia - Celebes (McDermott 1966, Ballantyne et al. 2016, Ballantyne et al. 2019).
20	*Luciolapallidipes* Pic, 1928 *	Sikkim; West Bengal - Kurseong (Ballantyne et al. 2019).	Brunei; Indonesia; Malayasia (Ballantyne et al. 2019). Malayasia - Malacca (McDermott 1966).
21	*Luciolasudra* Gorham 1903	India (?) (McDermott 1966)	
22	*Luciolatenuicornis* Olivier, 1885 **	India (?) (Ballantyne et al. 2016, Ballantyne et al. 2019)	Indonesia - Celebes, Kandari (Olivier 1885). New Guinea; Indonesia - Celebes (McDermott 1966). Indonesia (Ballantyne et al. 2016, Ballantyne et al. 2019).
23	*Luciolatincticollis* Gorham, 1895 ^##^	Karnataka - Belgaum (Gorham 1895). Inde (?) (Olivier 1905). India (?) (McDermott 1966, Ballantyne et al. 2016, Ballantyne et al. 2019).	
24	*Luciolatrivandrensis* Raj, 1947 ^##^	India (?) (Raj 1947, McDermott 1966, Ballantyne et al. 2019).	
25	*Luciolaxanthura* Gorham, 1880 **	Karnataka - Nilgiri hills; Kerala; Tamil Nadu (Gorham 1880, Olivier 1905, McDermott 1966). India (?) (Ballantyne et al. 2019).	
26	*Pteroptyxmalaccae* (Gorham, 1880)	Tamilnadu - Chennai (McDermott 1966, Ballantyne and McLean 1970, Ballantyne et al. 2011). West Bengal - herein.	Malaysia - Malacca; Indonesia - Sumatra (McDermott 1966, Olivier 1905). Borneo; Cambodia; Indonesia; Malaysia; Thailand (Ballantyne et al. 2019).
27	*Pygoluciolacalceata* (Olivier, 1905)	Pondicherry (Olivier 1905). India (?) (McDermott 1966). Pondicherry; Tamilnadu (Ballantyne et al. 2019).	Sri Lanka (Ballantyne et al. 2019).
28	*Pygoluciolainsularis* (Olivier, 1883)	Andaman Islands (Olivier 1905, McDermott 1966). Andaman Islands; Nicobars (Ballantyne et al. 2019).	Indonesia - Java, Sumatra (Ballantyne et al. 2019).
29	*Pygoluciolanitescens* (Olivier, 1903)	West Bengal - Darjeeling (Olivier 1905, McDermott 1966, Ballantyne et al. 2019).	Malaysian Borneo (Ballantyne et al. 2019). Borneo (Olivier 1905, McDermott 1966).
30	*Pygoluciolavitalisi* (Pic, 1934)	West Bengal - Kalimpong (Ballantyne et al. 2019).	Cambodia (McDermott 1966).
31	*Pyrophanessemilimbata* (Olivier 1883)	Indes Orientales (Olivier 1883, Gorham 1903, Olivier 1905). Eastern India (McDermott 1966). India (?) (Thancharoen et al. 2007).	Indonesia; Philippines; Malayasia (Ballantyne et al. 2015). Only the name mentioned (Ballantyne et al. 2019).
32	*Sclerotiaaquatilis* (Thancharoen, 2007)	India (?) (Ballantyne et al. 2016, Ballantyne et al. 2019). West Bengal (Ghosh et al. 2021). Odisha - herein.	Reports of some uncertain cases from Africa (Jeng et al. 2003, Thancharoen et al. 2007). Vietnam - Cochin China; Thailand (Ballantyne et al. 2016, Ballantyne et al. 2019).
33	*Sclerotiasubstriata* (Gorham, 1880)	Assam; Karnataka - Belgaum; Maharashtra - Mumbai (Gorham 1880, Gorham 1895). Assam - Guwahati; Maharashtra - Mumbai (Ballantyne et al. 2016, Ballantyne et al. 2019). West Bengal - herein.	Myanmar - Burma, Rangoon (Gorham 1880, Gorham 1895). Myanmar - Tharawaddy, Rangoon; Sri Lanka (Ballantyne et al. 2016, Ballantyne et al. 2019).

## References

[B8276638] Agarwal V. C., Ghose R. K. (1995). Fauna of Palamau Tiger Reserve. Fauna of Tiger Reserve (Sunderbans, Manas, Palamau, Simlipal). Zoological Survey of India. Fauna of Conservation Area Series.

[B8276647] Agarwal V. C., Ghose R. K. (1995). Fauna of Simlipal Tiger Reserve. Fauna of Tiger Reserve (Sunderbans, Manas, Palamau, Simlipal). Zoological Survey of India. Fauna of Conservation Area Series.

[B8276656] Agarwal V. C., Ghose R. K. (1995). Fauna of Sunderbans Tiger Reserve. Fauna of Tiger Reserve (Sunderbans, Manas, Palamau, Simlipal). Zoological Survey of India. Fauna of Conservation Area Series.

[B8451904] Ballantyne L., Kawashima I., Jusoh W. F.A., Suzuki H. (2022). A new genus for two species of Japanese fireflies having aquatic larvae (Coleoptera, Lampyridae) and a definition of *Luciola* s str.. European Journal of Taxonomy.

[B8276779] Ballantyne L. A., McLean M. R. (1970). Revisional studies on the firefly genus *Pteroptyx* Olivier (Coleoptera: Lampyridae: Luciolinae: Luciolini). Transactions of the American Entomological Society.

[B8276715] Ballantyne L. A., Lambkin C. L. (2009). Systematics of Indo-Pacific fireflies with a redefinition of Australasian *Atyphella* Olliff, Madagascan *Photuroluciola* Pic and description of seven new genera from the Luciolinae (Coleoptera: Lampyridae). Zootaxa.

[B8276696] Ballantyne L. A., Fu X. H., Shih C. H., Cheng C. Y., Vor Y. (2011). Pteroptyx
maipo Ballantyne, a new species of bent-winged firefly (Coleoptera: Lampyridae) from Hong Kong, and its relevance to firefly biology and conservation. Zootaxa.

[B8276724] Ballantyne L. A., Lambkin C. L. (2013). Systematics and phylogenetics of Indo-Pacific Luciolinae fireflies (Coleoptera: Lampyridae) and the description of new genera. Zootaxa.

[B8276683] Ballantyne L. A., Fu X. H., Lambkin C. L., Jeng M. L., Faust L., Wijekoon W. M.C.D., Daiqin L., Zhu T. (2013). Studies on South-east Asian fireflies: *Abscondita*, a new genus with details of life history, flashing patterns and behaviour of *Abs.chinensis* (L.) and *Abs.terminalis* (Olivier) (Coleoptera: Lampyridae: Luciolinae). Zootaxa.

[B8276733] Ballantyne L. A., Lambkin C. L., Boontop Y., Jusoh W. F.A. (2015). Revisional studies on the Luciolinae (Coleoptera: Lampyridae): 1. The firefly genus *Pyrophanes* Olivier with two new species. 2. Four new species of *Pteroptyx* Olivier. 3. *Inflata* gen. nov. Boontop, with redescription of *Luciolaindica* (Motsch.) as *Inflataindica* comb. nov. Zootaxa.

[B8276742] Ballantyne L. A., Jusoh W. F.A. (2016). List of genera and species in the Luciolinae from SE Asia and the Australopacific Version 2. Checklist of IndoPacific Luciolinae.

[B8276751] Ballantyne L. A., Lambkin C. L., Luan X., Boontop Y., Nak Eiam S., Pimpasalee S., Silalom S., Thancharoen A. (2016). Further studies on south eastern Asian Luciolinae: 1. *Sclerotia* Ballantyne, a new genus of fireflies with back swimming larvae. 2. *Triangulara* Pimpasalee, a new genus from Thailand (Coleoptera: Lampyridae). Zootaxa.

[B8276764] Ballantyne L. A., Lambkin C. L., Ho J. Z., Jusoh W. F.A., Nada B., Nak Eiam S., Thancharoen A., Wattanachaiyingcharoen W., Yiu V. (2019). The Luciolinae of S. E. Asia and the Australopacific region: a revisionary checklist (Coleoptera: Lampyridae) including description of three new genera and 13 new species. Zootaxa.

[B8276788] Bocakova M., Campello-Gonçalves L., Da Silveira L. F.L. (2022). Phylogeny of the new subfamily Cladodinae: neotenic fireflies from the Neotropics (Coleoptera: Lampyridae). Zoological Journal of the Linnean Society.

[B8276824] Branham M. A., Leschen R. A.B., Beutel R. G., Lawrence J. F. (2010). Coleoptera, Beetles. Volume 2: Morphology and Systematics (Elateroidea, Bostrichiformia, Cucujiformia partim).

[B8276934] Chen T. R. (2003). The fireflies of Taiwan.

[B8276942] Das A. K., Dev Roy M. K. (1989). A general account of the mangrove fauna of Andaman and Nicobar Islands. Zoological Survey of India: Fauna of Conservation Areas.

[B8276959] Ferreira V. S., Keller O., Branham M. A., Ivie M. A. (2019). Molecular data support the placement of the enigmatic Cheguevaria as a subfamily of Lampyridae (Insecta: Coleoptera). Zoological Journal of the Linnean Society.

[B8276968] Ferreira V. S., Keller O., Branham M. A. (2020). Multilocus phylogeny support the non-bioluminescent firefly Chespirito as a new subfamily in the Lampyridae (Coleoptera: Elateroidea). Insect Systematics and Diversity.

[B8277035] Fu X. H., Wang Y., Lei C., Nobuyoshi O. (2005). The swimming behavior of the aquatic larvae of the Firefly *Luciolasubstriata* (Coleoptera: Lampyridae). The Coleopterists Bulletin.

[B8277021] Fu X. H., Ballantyne L. A., Meyer-Rochow V. (2009). Bioluminescence in Focus, A Collection of illuminating essays.

[B8277012] Fu X. H., Ballantyne L., Lambkin C. (2012). *Emeia* gen. nov., a new genus of Luciolinae fireflies from China (Coleoptera: Lampyridae) with an unusual trilobite-like larva, and a redescription of the genus *Curtos* Motschulsky. Zootaxa.

[B9195867] Fu X. H., Ballantyne L., Lambkin C. (2012). The external larval morphology of aquatic and terrestrial Luciolinae fireflies (Coleoptera: Lampyridae). Zootaxa.

[B8277004] Fu X. H. (2014). An illustrated handbook of Chinese fireflies.

[B8288805] Ghosh S., Sarkar S. K., Chakraborty S. K. (2021). Two new records of the subfamily Luciolinae Lacordaire, 1857 (Coleoptera: Lampyridae) with a checklist of genus *Abscondita* from India. Journal of Asia Pacific Biodiversity.

[B8290078] Gorham H. S. (1880). Materials for a revision of the Lampyridae. Transactions of Entomological Society of London.

[B8290128] Gorham H. S. (1895). List of the Coleoptera in the collection of H. E. Andrewes Esq. from India and Burma, with descriptions of new species and notes. Annales de la Société Entomologique de Belgique.

[B8290146] Gorham H. S. (1903). On Coleoptera collected in India by MM. H. E. and H. L. Andrewes. Entomologique de Belgique.

[B8452816] Jeng M. L., Yang P. S., Satô M., Lai J., Chang J. C. (1998). The genus *Curtos* (Coleoptera, Lampyridae, Luciolinae) of Taiwan and Japan. Japanese Journal of Systematic Entomology.

[B8290311] Jeng M. L., Yang P. S., Lai J. (2003). Notes on the genus *Luciola* (Coleoptera, Lampyridae, Luciolinae) of Taiwan. Special Bulletin of the Japanese Society of Coleopterology.

[B8290404] Jusoh W. F.A., Ballantyne L. A., Lambkin C. L., Hashim N. R., Wahlberg N. (2018). The firefly genus *Pteroptyx* Olivier revisited (Coleoptera: Lampyridae: Luciolinae. Zootaxa.

[B8290389] Jusoh W. F., Ballantyne L. A., Chan S. H., Wong T. W., Yeo D., Nada B., Chan K. O. (2021). Molecular systematics of the firefly genus *Luciola* (Coleoptera: Lampyridae: Luciolinae) with the description of a new species from Singapore. Animals.

[B8290422] Kacker R. K. (1993). Chromosomes and phylogeny of Coleoptera. Records of the Zoological Survey of India.

[B8290431] Kapur A. P. (1955). Contribution to a knowledge of the fauna of Manipur state, Assam. v. Coleoptera. Records of the Zoological Survey of India.

[B8290962] Lloyd J. E., Wing S. R., Hongtrakul T. (1989). Ecology, flashes and behavior of congregating Thai fireflies. Biotropica.

[B8291327] Majumder S. C., Dey A. (2005). Studies on some ethnomedicinal arachnids and insects in relation to their usage as drugs among the tribals of Sundarbans, West Bengal, India. Records of Zoological Survey of India.

[B8291345] Martin G. J., Branham M. A., Whiting M. F., Bybee S. M. (2017). Total evidence phylogeny and the evolution of adult bioluminescence in fireflies (Coleoptera: Lampyridae).. Molecular Phylogenetics and Evolution.

[B8291354] Martin G. J., Stanger-Hall K. F., Branham M. A., Silveira L. F.L., Lower S. E., Hall D. W., Li X. -Y., Lemmon A. R., Moriarty Lemmon E., Bybee S. M. (2019). Higher-level phylogeny and reclassification of Lampyridae (Coleoptera: Elateroidea). Insect Systematics and Diversity.

[B9220579] McDermott F. A. (1964). The taxonomy of the Lampyridae. Transactions of the American Entomological Society.

[B8291396] McDermott F. A., Steel W. O. (1966). Coleopterorum Catalogus Supplementa. Pars 9.

[B8291418] Mitra T. R. (2005). Taxonomic assessment of insects recorded in Kalidasa’s works. Rec. zool. Surv. India.

[B8296952] Motschulsky V. (1854). Lampyrides. Etudes Entomologiques.

[B8291495] Nak-eiam S., Wattanachaiyingcharoen W., Thancharoen A. (2011). Distribution and habitat of the firefly, *Asymmetricatacircumdata* (Motsch.) (Coleoptera: Lampyridae: Luciolinae) in the North of Thailand. NU Science Journal.

[B8291535] Olivier E. (1883). Lampyrides nouveaux oupeuconnus. Revue D’Entomologie.

[B8291544] Olivier E. (1885). Catalogue des Lampyrides faisant partie des collections du Musée Civique de Gênes. Annali del Museo Civico di Storia Naturale di Genova.

[B8291608] Olivier E. (1905). Descriptions de Lampyrides nouveaux. Annales de la Société Entomologique de Belgique.

[B8452807] Pronak K., Khongcharoen P., Poolprasert P. (2018). A wide range of fireflies (Coleoptera: Lampyridae) in Phitsanulok Province. YRU Journal of Science and Technology.

[B8293280] Rabha M. M., Gohain Barua A. (2016). Bioluminescence emissions of female fireflies of the species *Asymmetricatacircumdata*. Asian Journal of Physics.

[B8293289] Raj J. S. (1947). Two species of undescribed Lampyrid larvae from S India. Proceedings of the Indian Academy of Science.

[B8293315] Roy M., Nandi N. C. (2012). Insects. Fauna of Indian Museum Tank. (Ed. Director, Zoological Survey of India).. Records of the Zoological Survey of India.

[B8293342] Sengupta T., Mukhopadhyay P., Jairajpuri M. S. (1990). Collection and Preservation of Animals.

[B8293364] Seri N. A., Rahman A. A. (2021). Fireflies in South East Asia: Species Diversity, Distribution, and Habitat (2015-2021). Tropical Agricultural Science.

[B8293373] Thancharoen A., Ballantyne L. A., Branham M. A., Jeng M. L. (2007). Description of *Luciolaaquatilis* sp. nov., a new aquatic firefly (Coleoptera: Lampyridae: Luciolinae) from Thailand. Zootaxa.

[B8452799] Theraphat T. (1969). Study on life cycle, development and light producing organs in the firefly, *Luciolacircumdata* Mots.

[B8293391] Wattanachaiyingcharoen W., Nak-Eiam S., Phanmuangma W., Booninkiaew S., Nimlob N. (2016). Species diversity of firefly (Coleoptera: Lampyridae) in the highlands of Northern Thailand. International Journal of Science.

[B8293401] Wijekoon W. M.C.D., Wegiriya H. C.E., Bogahawatha C. N.L. (2012). Regional diversity of fireflies of the subfamily Luciolinae (Coleoptera: Lampyridae) in Sri Lanka. Lampyrid.

[B8293410] Wijekoon W. M.C.D., Wegiriya H. C.E., Bogahawatha C. N.L. (2016). Systematic revision of the repository collection of Canthoroidea (sic) in the Department of National Museums, Colombo, Sri Lanka (Coleoptera: Cantharidae, Lampyridae, Lycidae, Rhagophthalmidae. Ceylon Journal of Science.

[B8293419] Wijekoon W. M., Wegiriya H., Bogahawatha C. (2021). Distribution, Diversity and Relative Abundance of Fireflies (Coleoptera; Lampyridae) in Three Habitat Types in Sri Lanka. Rajarata University Journal.

[B8293450] Yiu V. (2012). Fireflies of Hong Kong. Popular Entomology Book Series No. 7..

[B8293467] Zheng X., Fu X., Zhang S., Lei C. (2008). Larval behaviour probably associated with respiration in *Luciolasubstriata* Gorham (Coleoptera: Lampyridae). The Coleopterists Bulletin.

